# Strong intracellular signal inactivation produces sharper and more robust signaling from cell membrane to nucleus

**DOI:** 10.1371/journal.pcbi.1008356

**Published:** 2020-11-16

**Authors:** Jingwei Ma, Myan Do, Mark A. Le Gros, Charles S. Peskin, Carolyn A. Larabell, Yoichiro Mori, Samuel A. Isaacson

**Affiliations:** 1 Department of Mathematics and Statistics, Boston University, Boston, Massachusetts, United States of America; 2 Department of Cellular and Molecular Medicine, University of California, San Diego Medical School, San Diego, California, United States of America; 3 Department of Anatomy, University of California, San Francisco, San Francisco, California, United States of America; 4 National Center for X-ray Tomography, Lawrence Berkeley National Lab, Berkeley, California, United States of America; 5 Courant Institute of Mathematical Sciences, New York University, New York, New York, United States of America; 6 Department of Mathematics, University of Pennsylvania, Philadelphia, Pennsylvania, United States of America; University of Pittsburgh, UNITED STATES

## Abstract

For a chemical signal to propagate across a cell, it must navigate a tortuous environment involving a variety of organelle barriers. In this work we study mathematical models for a basic chemical signal, the arrival times at the nuclear membrane of proteins that are activated at the cell membrane and diffuse throughout the cytosol. Organelle surfaces within human B cells are reconstructed from soft X-ray tomographic images, and modeled as reflecting barriers to the molecules’ diffusion. We show that signal inactivation sharpens signals, reducing variability in the arrival time at the nuclear membrane. Inactivation can also compensate for an observed slowdown in signal propagation induced by the presence of organelle barriers, leading to arrival times at the nuclear membrane that are comparable to models in which the cytosol is treated as an open, empty region. In the limit of strong signal inactivation this is achieved by filtering out molecules that traverse non-geodesic paths.

## Author summary

The inside of cells is a complex spatial environment, filled with organelles, filaments and proteins. It is an open question how cell signaling pathways function robustly in the presence of such spatial heterogeneity. In this work we study how organelle barriers influence the most basic of chemical signals; the diffusive propagation of an activated protein from the cell membrane to nucleus. Three-dimensional B cell organelle and membrane geometries reconstructed from soft X-ray tomographic images are used in building mathematical models of the signal propagation process. Our models demonstrate that organelle barriers significantly increase the time required for a diffusing protein to traverse from the cell membrane to nucleus when compared to a cell with an empty cytosolic space. We also show that signal inactivation, a fundamental component of all signaling pathways, can provide robustness in the signal arrival time in two ways. Increasing rates of signal inactivation reduce variability in the arrival time, while also dramatically reducing the degree to which organelle barriers increase the arrival time (in comparison to a cell with an empty cytosol).

## Introduction

Spatial dynamics can play a critical role in the successful functioning of cellular signaling processes, where as basic a property as cell shape can significantly influence the behavior of signaling pathways [1, 2]. Idealized one-dimensional [3], spherical [2, 4, 5] or planar [6] geometries are commonly used in mathematical models of the cell, with the cytosol represented as an empty region of fluid [1–3]. Despite the simplicity of the representation of the plasma membrane and/or cytosolic space, the study of spatial signaling dynamics within mathematical models has provided key insights into the function of many biological pathways, including cyclic AMP signaling in neurons [1], T cell synapse formation through T cell receptor signaling [6], B cell activation through kinase-receptor interactions [4], and general protein kinase signaling [2, 3, 5]. For example, changes in idealized cell shapes can induce significant changes in the timing of signal propagation and the size of concentration gradients across the cytosol [2].

**Fig 1:**
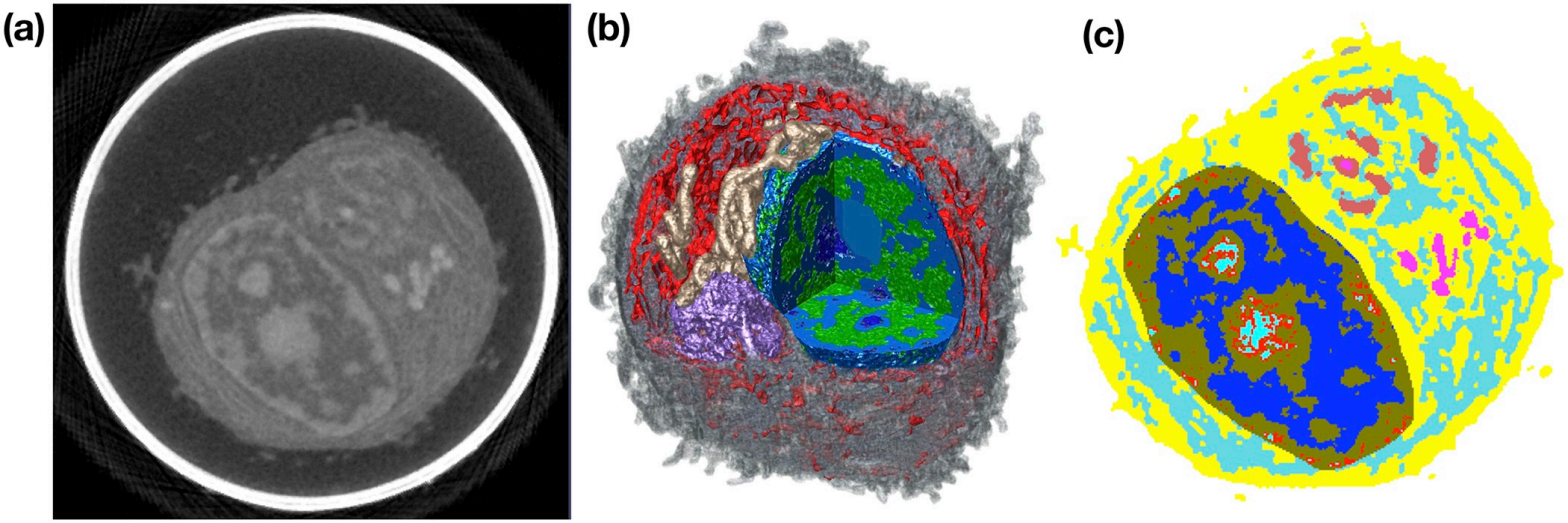
Soft X-ray tomography (SXT) imaging of human B cells. **(a)** One 2D image plane within a 3D SXT reconstruction of a B cell. The corresponding 3D reconstruction is subsequently labeled as Bcell1 in simulations. Pixel intensity corresponds to linear absorption coefficient (LAC), a measure of the local density of organic material [10, 11]. Larger LAC values are shown in lighter colors. The bright white band corresponds to the glass capillary in which the cryo-preserved cell was contained. **(b)** 3D SXT reconstruction of a human B cell with cutaway to show segmented organelles: heterochromatin (blue), euchromatin (green), mitochondria (beige), Golgi (purple) and endoplasmic reticulum (ER) (red). Bulk cytosol is shown in gray, with the cell membrane given by the outer boundary of the cytosol. In our mathematical model, the nucleus, *N*, is given by the set of voxels with labels corresponding to components of the nucleus (e.g. euchromatin and heterochromatin in this image). Cytosol, *C*, is given by voxels rendered in gray, while all other (colored) voxels outside the nucleus are labeled as organelles, *O*. **(c)** Organelle label field values for voxels within the cell in the image plane shown in (a). Here free cytosolic space corresponds to the regions in yellow, and voxels outside the cell are not shown.

In modeling signal propagation from the cell membrane to the nucleus, a further challenge arises from the crowded, spatially heterogeneous nature of the cytosolic space [7]. In this work we investigate the question of how spatial heterogeneity arising from organelle barriers, as illustrated in Fig 1b, might influence the propagation of signals from the cell membrane to the nuclear membrane. We consider the simplest possible model for signal propagation from the cell membrane to the nucleus, the release of a one or more activated proteins from the inner cell membrane, and their diffusion throughout the cytosol until they first reach the nuclear membrane. As the classical picture of signal propagation to the nucleus typically involves large pathways of many chemically reacting molecules (such as the MAPK pathway [3]), this model may seem overly simplified. However, a number of proteins are known to be activated at the cell membrane and then directly translocate to the nucleus [8, 9]. For example, in Notch signaling the extracellular domain of Notch receptor can interact with ligands, leading to release of NICD (Notch intracellular domain) from the plasma membrane into the cytosol. NICD then translocates to the nucleus where it can regulate gene transcription [8, 9]. More generally, studying signals that correspond to the diffusive propagation from cell membrane to nucleus of individual proteins provides a first step towards understanding how cellular substructure might influence the dynamics of more complicated signaling pathways.

Using segmented reconstructions of organelle geometry obtained by soft X-ray tomography (SXT) imaging, we study how the presence of organelle barriers modifies the time needed for diffusing molecules to reach the nucleus in comparison to the time required within an empty cytosol. As signaling molecules diffusing through the cytosol can not persist indefinitely, we next investigate how signal inactivation might influence the search process. This creates a competition where the diffusing signal may be inactivated or degraded prior to reaching the nuclear membrane. We study how the strength of signal inactivation can modulate statistics of the first passage time (FPT) for an individual molecule to reach the nucleus, conditional on it reaching the nucleus before inactivation. It is shown that if the total signal (i.e. number of molecules) that ultimately reach the nucleus is held constant, increasing the inactivation rate leads to signal sharpening. We also find that signal inactivation can provide robustness to the presence of organelle barriers, significantly reducing the difference between the average arrival time of molecules that successfully reach the nucleus in geometries containing organelle barriers, from the time in geometries containing an empty cytosol.

We note that our studies focus on statistics of the time required for the diffusing protein to reach the nucleus. In the case that there is no inactivation, so that the protein simply diffuses until reaching the nucleus, this is an example of a classical diffusion-limited first passage time problem [12]. First passage time problems are widely used in the study of chemical reactions [13, 14], with a variety of asymptotic results and exact solution techniques when the target site is small or a basic geometrical shape such as a sphere [15–18].

## Results

### Mathematical model

We consider the time required for a protein to diffuse from the cell membrane to the nuclear membrane. Let *N* denote the nucleus of the cell, with ∂*N* denoting the nuclear membrane. Similarly, we let *C* denote the cytosol of the cell, with ∂*C* denoting the cell membrane. We assume the cytosol may be filled with a collection of closed subvolumes corresponding to organelles, denoted by *O*, with boundary surfaces ∂*O*. Fig 1a shows a slice plane through a 3D soft X-ray tomography (SXT) reconstruction of a human B cell illustrating such geometries, with Fig 1b showing a 3D reconstruction identifying the nucleus, cytosolic organelles, and the cytosol.

We assume a molecule is initially activated at the cell membrane, and diffuses throughout the cytosolic space until it first reaches the nuclear membrane. Both the cell membrane and organelle surfaces are assumed to be reflecting barriers to the molecule’s diffusion. Denote by *D* = 10(*μ*m)^2^s^−1^ the diffusivity of the molecule, and by *p*(***x***, *t*) the probability density the molecule is located at position ***x*** within *C* at time *t*. ***η***(***x***) will denote the unit outward normal to a surface at ***x***. *p*(***x***, *t*) then satisfies the diffusion equation

$$\begin{array}{c} \begin{array}{cccc} \frac{\partial p}{\partial t}\left(x,t\right) & =D\Delta p(x,t), & & x\in C,\\ p(x,t) & =0, & & x\in \partial N,\\ \nabla p(x,t)\cdot \eta (x) & =0, & & x\in \partial O\;\text{or}\;\partial C,\\ p(x,0) & =g(x), & & x\in C\cup \partial C. \end{array} \end{array}$$ (1)

Note, in the following we assume the initial position of the molecule is located on the inner surface of the cell membrane, so that *g*(***x***) is zero away from ∂*C*. The Dirichlet boundary condition on ∂*N* in (1) encodes that the protein is instantly absorbed upon reaching the nuclear membrane, allowing us to study statistics of diffusing protein’s arrival time at the nuclear membrane.

Let *T* denote the random time at which the protein first reaches the nuclear membrane surface. The survival probability that the protein has not yet reached ∂*N* at time *t* is then given by

$$S\left(t\right)=\mathrm{Prob}[T>t]=\int_{C}p\left(x,t\right)\;dx.$$

The corresponding probability per time the molecule reaches ∂*N* is the probability density function (pdf)

$$\begin{array}{c} f(t)=-\frac{dS}{dt}=-D\int_{\partial N}\nabla p(x,t)\cdot \eta (x)dA(x), \end{array}$$ (2)

where *dA*(***x***) denotes the surface area measure at ***x*** ∈ ∂*N*. Knowing *f*(*t*), we can calculate statistics of *T*, using that the average of a function *w*(*T*), denoted by E[w(T)], is defined by

$$E[w(T)]=\int_{0}^{\infty }w\left(t\right)f\left(t\right)\;dt.$$

Our representations of cellular geometry are derived from 3D SXT reconstructions, see Methods, for which the label field identifying organelles is provided as a Cartesian grid of cubes with mesh-width *h*, see Fig 1. To simulate the time required for the protein to traverse the cytosol we therefore discretize (1) onto this grid, generating a system of ODEs we solve numerically. Let *C*_*h*_ denote the collection of mesh voxels that are labeled as being cytosol, with *N*_*h*_ those that are labeled as being within the nucleus, and *O*_*h*_ those within organelles. We label the individual voxels within the cytosol by $C_{h}=\{V_{i}{\}}_{i=1}^{M}$, and let $N(V_{i};C_{h})$ denote the indices of the subset of the six Cartesian grid nearest-neighbors of voxel *V*_*i*_ that are *within* the cytosol. $N(V_{i};N_{h})$ will similarly denote the indices of the subset of the six Cartesian grid nearest-neighbors of *V*_*i*_ that are within the nucleus. For ***x***_*i*_ denoting the centroid of voxel *V*_*i*_, we let *p*_*h*_(***x***_*i*_, *t*) ≈ *p*(***x***_*i*_, *t*). *p*_*h*_ then satisfies the semi-discrete diffusion equation that

$$\begin{array}{c} \begin{array}{cccc} \frac{dp_{h}}{dt}(x_{i},t) & =D(\Delta_{h}p_{h})(x_{i},t), & & V_{i}\in C_{h}\\ p_{h}(x_{i},0) & =g_{h}(x_{i}), & & V_{i}\in C_{h}, \end{array} \end{array}$$ (3)

where the discrete Laplacian is defined by

$$\begin{array}{c} \begin{array}{cc} \left(\Delta_{h}p_{h}\right)\left(x_{i},t\right)=\frac{1}{h^{2}}[ & \sum_{j\in N(V_{i};C_{h})}(p_{h}\left(x_{j},t\right)-p_{h}\left(x_{i},t\right))-\sum_{j\in N(V_{i};N_{h})}p_{h}\left(x_{i},t\right)], \end{array} \end{array}$$ (4)

and *g*_*h*_(***x***_*i*_) denotes the initial condition in the semi-discrete model.

This semi-discrete model corresponds to approximating the continuous Brownian motion of the particle in *C* by a continuous-time random walk of the molecule hopping between nearest-neighbor voxels of *C*_*h*_.

If we denote by *T*_*h*_ the corresponding random time for the protein to first reach a voxel that is labeled as being within the nucleus, we have the corresponding survival probability,

$$S_{h}\left(t\right)=\mathrm{Prob}[T_{h}>t]=\sum_{V_{i}\in C_{h}}p\left(x_{i},t\right)h^{3},$$

with analogous definitions for the pdf *f*_*h*_(*t*) and averages, $E\left[w(T_{h})\right]$, as above.

In the remainder, unless stated otherwise time will be reported in units of seconds, and distance in units of *μ*m.

### Organelle barriers slow the propagation of a signal from the cell membrane to nucleus, while increasing variability in arrival time for signals initiated at different locations

We begin by numerically solving (3) to investigate how the presence of organelles as reflecting barriers influences statistics of the time required for the diffusing protein to reach the nuclear membrane. Let ∂*C*_*h*_ denote the collection of voxels within the free cytosol, *C*_*h*_, that border the exterior of the cell, with |∂*C*_*h*_| denoting the volume of this set of voxels. Note, this collection of voxels corresponds to a thin region of cytosol bordering the cell membrane. In the semi-discrete model, we will approximate starting the protein uniformly distributed on the inner surface of the cell membrane by starting the protein uniformly within the volume ∂*C*_*h*_. Then

$$\begin{array}{c} g_{h}(x_{i})=\{\begin{array}{cc} \frac{1}{\left|\partial C_{h}\right|}, & V_{i}\in \partial C_{h},\\ 0, & \text{else}. \end{array} \end{array}$$ (5)

**Fig 2:**
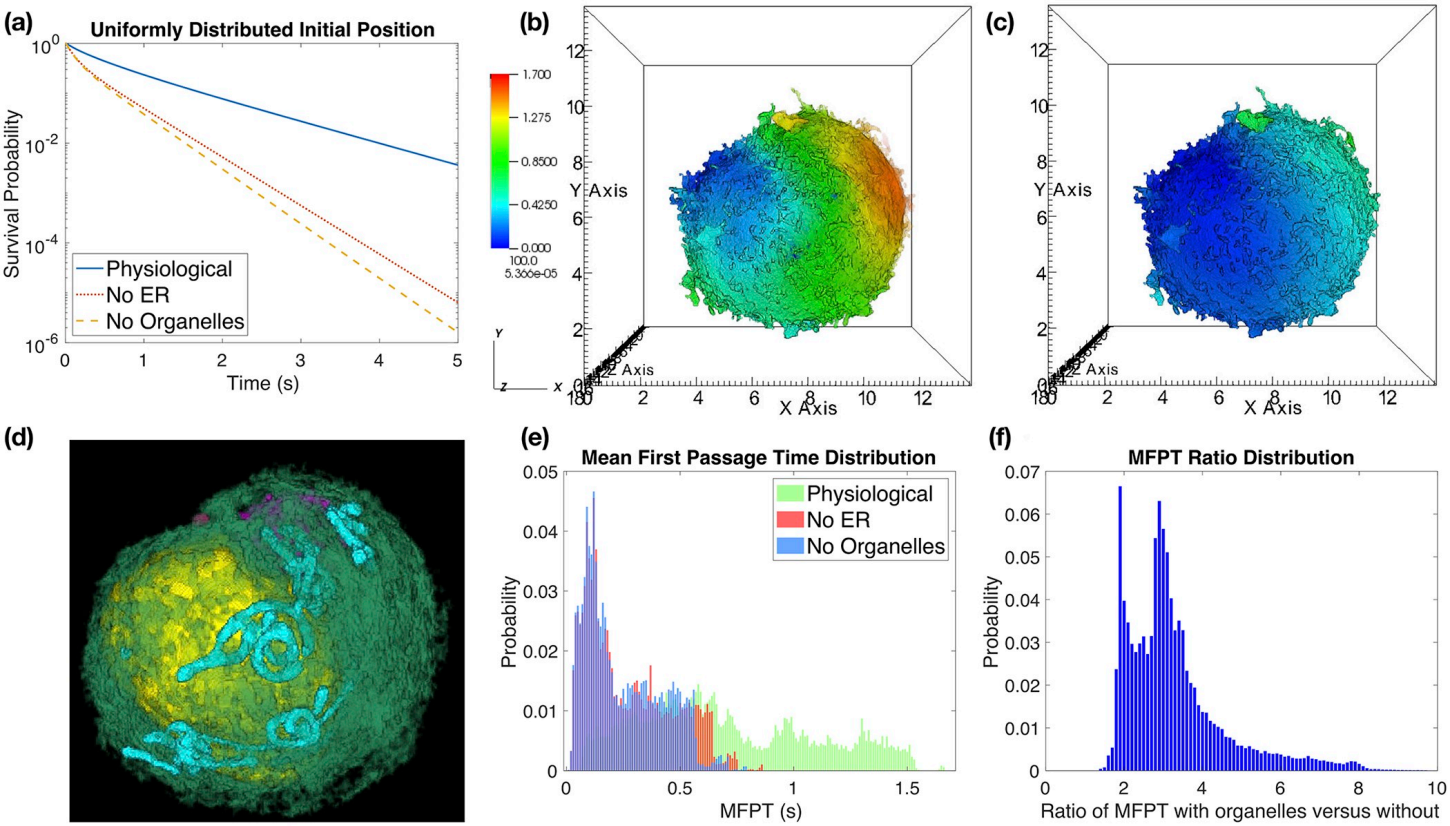
The presence of organelles as diffusive barriers increases the time required for a diffusing (signaling) molecule to traverse from the cell membrane to the nuclear membrane. **(a)** Survival probability, *S*_*h*_(*t*), when the diffusing molecule is started uniformly distributed within a thin region, ∂*C*_*h*_, of cytosol bordering the inner surface of the cell membrane (5). **(b)** Mean first passage time (MFPT) *u*(***x***_*i*_) from each voxel within ∂*C*_*h*_ to reach the nuclear membrane in the “physiological” case that organelles are present as diffusive barriers. Colorbar gives the MFPT values in seconds, spatial units are *μ*m. **(c)** Corresponding MFPTs in the “no organelles” case that the molecules can freely diffuse everywhere between the cell and nuclear membranes. Color scale is the same as (b). **(d)** Volume rendering of the organelles in Bcell1, with the cell in the same orientation as in (b) and (c) (but zoomed in). Note, the ER rendering (green) is attenuated to make other organelles more apparent, and the cell membrane is not shown. Nucleus is in yellow, mitochondria in cyan, and the Golgi in purple. **(e)** Distributions of mean first passage times (MFPTs), $\{u_{h}(x_{i}){\}}_{V_{i}\in \partial C_{h}}$, starting from the same thin region of cytosolic voxels bordering the cell membrane as in (b) and (c). Note, here the distribution is over the voxels within the region, illustrating how starting at different initial positions can lead to variation in the MFPT. For the “No ER” case we use the analogous region when just the ER is removed. See (6) for definition of the MFPTs *u*_*h*_(***x***_*i*_). Bin width is .01 (seconds). **(f)** Distribution of the ratios of the corresponding “Physiological” to “No Organelles” MFPTS from (e). This illustrates when starting from each individual voxel bordering the cell membrane, how much organelle barriers increase the MFPT to reach the nucleus from that voxel. Bin width is .1. Note, almost all locations have a ratio of two or more, showing that organelle barriers significantly increase the time required to reach the nuclear membrane from most initial positions. Fig A and Fig B of S1 Text show similar results for Bcell2 and Bcell3 respectively. The obscured z-axes labels in panels (b) and (c) range from zero to eighteen on a linear scale.

In Fig 2a we show the survival probability *S*_*h*_(*t*) from Bcell1, the reconstruction shown in Fig 1 (results from two additional cell reconstructions, labeled Bcell2 and Bcell3, are shown in Fig A and Fig B of S1 Text). We consider three cases, the physiological data where voxels corresponding to organelles within the cytosol are inaccessible (labeled “physiological”), a modified geometry where voxels corresponding to the endoplasmic reticulum (ER) are added back into the collection of cytosolic voxels the protein can diffuse through (labeled “no ER”), and a modified geometry where all voxels within cytosolic organelles are added back into the collection of cytosolic voxels the protein can diffuse through (labeled “no organelles”). This latter geometry corresponds to the cytosol filling all space between the cell membrane and the nuclear membrane. In Fig 2a we observe that the presence of organelle barriers dramatically increases the time required for the protein to reach the nuclear membrane (shifting the survival probability curve upwards), with the primary contribution to this shift arising from the barrier provided by the ER. Table 1 shows that the corresponding mean and median times to reach the cell membrane change similarly. For Bcell1, the presence of the ER as a barrier accounts for most of the the time required to reach the nucleus; removing the ER decreases the median of *T*_*h*_ by almost a factor of three.

**Table 1:** Statistics of *T*_*h*_, the random time to reach the nucleus in Bcell1. The diffusing molecule is assumed to initially be randomly distributed on the cell membrane, ∂*C*_*h*_. Here STD denotes standard deviation and CV denotes the coefficient of variation (the standard deviation divided by the mean). Values in parenthesis denote the ratio of the physiological value to the corresponding no ER or no organelle values. See Table A of S1 Text for statistics in Bcells 2 and 3.

	Physiological	No ER	No Organelles
Bcell1 Mean	0.7070	0.2721 (2.6)	0.2499 (2.8)
Bcell1 Median	0.4054	0.1393 (2.9)	0.1335 (3.0)
Bcell1 STD	0.8472	0.3561	0.3173
Bcell1 CV	1.1983	1.3086	1.2695

In Fig 2b–2f we examine how the time to reach the nucleus varies when the diffusing molecule is started at different points on the cell membrane. Let *u*(***x***) denote the mean first passage time (MFPT) to diffuse from ***x*** ∈ *C* to the nuclear membrane. *u*(***x***) then satisfies [19]

$$\begin{array}{cccc} \Delta u(x) & =-\frac{1}{D}, & & x\in C\\ u(x) & =0, & & x\in \partial N\\ \nabla u(x)\cdot \eta (x) & =0, & & x\in \partial O\;\text{or}\;\partial C. \end{array}$$

In practice, we solve a discretized version of this PDE that gives the corresponding MFPTs on our Cartesian grid arising from the imaging data. Let *u*_*h*_(***x***_*i*_) denote the MFPT to reach the nucleus from ***x***_*i*_, which satisfies the linear system of equations

$$\begin{array}{c} \begin{array}{cccc} \left(\Delta_{h}u_{h}\right)\left(x_{i}\right) & =-\frac{1}{D}, & & V_{i}\in C_{h}. \end{array} \end{array}$$ (6)

Fig 2b plots *u*_*h*_(***x***_*i*_) over the cytosolic voxels bordering the cell membrane (∂*C*_*h*_) in the physiological case, while Fig 2c shows the case with no organelles (i.e. an empty cytosol). We see that the presence of organelles *significantly* slows the MFPT to the nucleus for most points bordering the cell membrane. Not surprisingly, locations closest to the nucleus (left side) generally have smaller MFPTs than locations far from the nucleus (right side). Fig 2e shows that the distribution of MFPTs, $\{u(x_{i}){\}}_{V_{i}\in \partial C_{h}}$, across the cytosolic voxels bordering the cell membrane is much flatter and broader when organelles are present as barriers (green, physiological case) in comparison to an empty cytosol (purple, no organelles case). Moreover, examining the ratio of these MFPTs in the physiological case to the no organelle case, Fig 2f, we find that at almost all locations the presence of organelle barriers increases the MFPT by a factor of two or more.

In conclusion, we observe that organelle barriers can substantially hinder the diffusion of molecules across the cytosol, significantly increasing the time required to reach the nuclear membrane, and increasing the variability of this time *over cytosolic voxels bordering the cell membrane* when comparing signals initiated at different points (Fig 2f). While our discussion has focused on Bcell1, we observe similar qualitative behavior in Bcell2 and Bcell3, see Fig A and Fig B of S1 Text.

### Inactivation filters out molecules undergoing longer searches, reducing variability in signal arrival time

Activated signaling molecules cannot diffuse throughout the cytosol of cells searching for the nuclear membrane indefinitely. Whether by degradation mechanisms, or inactivation mechanisms (such as phosphorylation or dephosphorylation), cellular signals will eventually be terminated. From the perspective of a diffusing signaling molecule this creates a competition between the search for the nuclear membrane and the inactivation process. We now examine how the interplay between these two processes can modulate the timing at which activated signals reach the cell membrane.

We consider the simplest possible mechanism for modeling signal inactivation, assuming the diffusing molecule can now also be inactivated with probability per time λ. Let *p*_λ_(***x***, *t*) denote the probability density the diffusing molecule is still activated and within the cytosol at time *t*. *p*_λ_ then satisfies

$$\begin{array}{c} \begin{array}{cccc} \frac{\partial p_{\lambda }}{\partial t}\left(x,t\right)=D\Delta p_{\lambda }\left(x,t\right) & -\lambda p_{\lambda }\left(x,t\right), & & x\in C,\\ p_{\lambda }\left(x,t\right) & =0, & & x\in \partial N,\\ \nabla p_{\lambda }\left(x,t\right)\cdot \eta \left(x\right) & =0, & & x\in \partial O\;\text{or}\;\partial C,\\ p_{\lambda }\left(x,0\right) & =g(x), & & x\in C\cup \partial C. \end{array} \end{array}$$ (7)

Note that *p*_λ_(***x***, *t*) = *e*^−*λt*^ *p*(***x***, *t*), so that *p*_0_(***x***, *t*) = *p*(***x***, *t*), the solution to the diffusion equation (1).

We are interested in statistics of the exit time through the nuclear membrane, *T*_λ_, conditioned on the protein actually reaching the nuclear membrane before inactivation (i.e. the event that *T*_λ_ < ∞). The probability per time that the diffusing molecule reaches the nuclear membrane at time *t* is then

$$\begin{array}{c} f_{\lambda }\left(t\right)=-D\int_{\partial N}\nabla p_{\lambda }\left(x,t\right)\cdot \eta \left(x\right)\;dA\left(x\right)=e^{-\lambda t}f\left(t\right) \end{array}$$ (8)

where *f*(*t*) = *f*_0_(*t*) denotes the probability per time to reach the nuclear membrane in the absence of degradation, given by (2). With these definitions, the probability the molecule reaches the nuclear membrane before inactivation is

$$Z_{\lambda }:=\mathrm{Prob}[T_{\lambda }<\infty ]=\int_{0}^{\infty }f_{\lambda }\left(t\right)\;dt=\int_{0}^{\infty }e^{-\lambda t}f\left(t\right)\;dt.$$

Denoting the conditional cumulative distribution function (CDF) of *T*_λ_ by

$$\begin{array}{c} F_{\lambda }\left(t\right)=\mathrm{Prob}[T_{\lambda }<t\;|\;T_{\lambda }<\infty ]=\frac{\int_{0}^{t}f_{\lambda }\left(s\right)\;ds}{\int_{0}^{\infty }f_{\lambda }\left(s\right)\;ds}, \end{array}$$ (9)

in Section SI1 of S1 Text we prove the following results

**Theorem 1** *For all fixed t* > 0 *and* λ ≥ 0, *Z*_λ_(*t*) *is a strictly decreasing function of* λ, *and F*_λ_(*t*) *is a strictly increasing function of* λ.

This result gives several immediate corollaries, including that

**Corollary 1** *Both the conditional MFPT*, $\langle T_{\lambda }\rangle ≔E[T_{\lambda }|T_{\lambda }<\infty ]$, *and the conditional median first passage time*, $M(T_{\lambda }):=F_{\lambda }^{-1}\left(\frac{1}{2}\right)$, *are strictly decreasing with respect to* λ. That 〈*T*_λ_〉 is decreasing in λ was also shown in [20] for probability density functions with the factored form *e*^−*λt*^ *g*(*t*).

Theorem 1 and Corollary 1 together demonstrate that as the inactivation rate λ is increased, the time for a molecule to reach the nucleus, *conditioned* on the molecule actually reaching the nucleus, decreases. The probability any individual molecule actually reaches the nucleus, *Z*_λ_, also decreases as λ increases. In this way strong signal inactivation will filter out molecules undergoing longer diffusive searches.

To explore how increasing the inactivation rate λ influences statistics of the time to reach the nucleus, we now study a semi-discrete model defined on the meshes representing the B cell geometries, and corresponding to a spatial discretization of (7). Let *p*_λ,*h*_(***x***_*i*_, *t*) ≈ *p*_λ_(***x***_*i*_, *t*) denote the probability density that the diffusing molecule is located at ***x***_*i*_ at time *t*, then

$$\begin{array}{c} \begin{array}{cccc} \frac{dp_{\lambda ,h}}{dt}(x_{i},t) & =D\left(\Delta_{h}p_{\lambda ,h}\right)\left(x_{i},t\right)-\lambda p_{\lambda ,h}\left(x_{i},t\right), & & V_{i}\in C_{h}\\ p_{\lambda ,h}\left(x_{i},0\right) & =g_{h}\left(x_{i}\right), & & V_{i}\in C_{h}, \end{array} \end{array}$$ (10)

where *p*_λ,*h*_(***x***_*i*_, *t*) = *e*^−*λt*^ *p*_*h*_(***x***_*i*_, *t*). Similarly, *f*_λ,*h*_(*t*) = *e*^−*λt*^ *f*_*h*_(*t*), so that the probability the diffusing molecule reaches the nucleus is given by

$$\begin{array}{c} Z_{\lambda ,h}=\int_{0}^{\infty }f_{\lambda ,h}\left(t\right)\;dt=\int_{0}^{\infty }e^{-\lambda t}f_{h}\left(t\right)\;dt. \end{array}$$ (11)

For *T*_λ,*h*_ the random time at which the nucleus is reached, the conditional MFPT to reach the nucleus is then

$$\begin{array}{c} \begin{array}{cc} \left\langle T_{\lambda ,h}\right\rangle & =E[T_{\lambda ,h}\;|\;T_{\lambda ,h}<\infty ]=-\frac{d}{d\lambda }\ln\left(Z_{\lambda ,h}\right)=-\frac{Z_{\lambda ,h}^{\prime }}{Z_{\lambda ,h}}=\frac{\int_{0}^{\infty }te^{-\lambda t}f_{h}\left(t\right)\;dt}{\int_{0}^{\infty }e^{-\lambda t}f_{h}\left(t\right)\;dt}. \end{array} \end{array}$$ (12)

**Fig 3:**
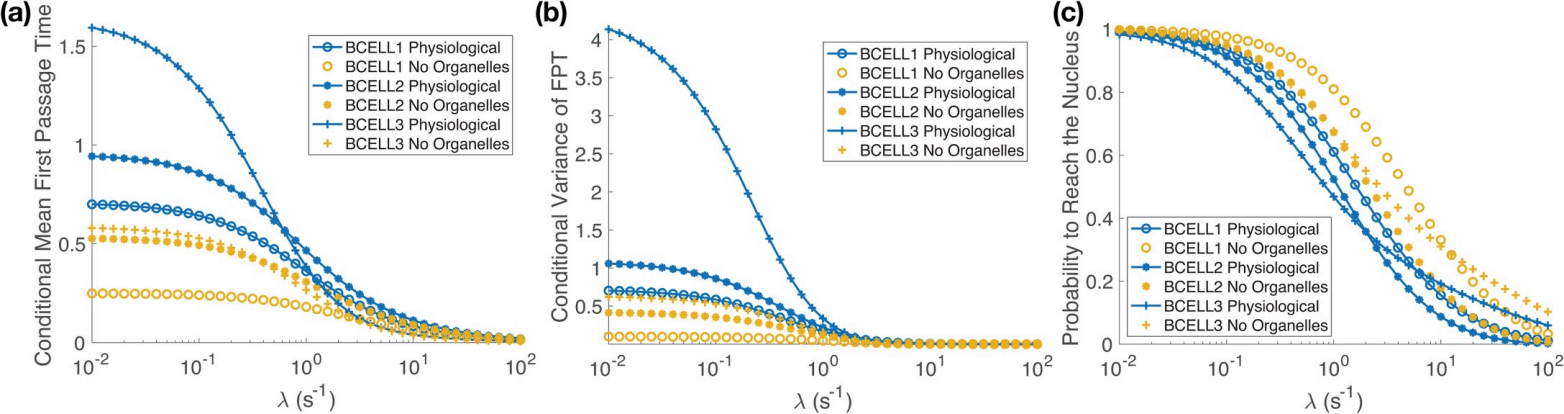
Signal inactivation filters out molecules undergoing longer diffusive searches, reducing both the average ime and variance in the time at which a molecule reaches nucleus, conditional on the molecule reaching the nucleus before inactivation. The figures show statistics of the conditional first passage time, *T*_λ,*h*_, to reach the nucleus when the diffusing molecule is started randomly on the cell membrane (i.e. uniformly distributed, see (5)), and the molecule can be inactivated with rate λ. **(a)** The conditional mean first passage time (MFPT), 〈*T*_λ,*h*_〉 (12). In all cases we see that 〈*T*_λ,*h*_〉 is strictly decreasing as λ increases, illustrating Corollary 1. Fig D of S1 Text shows an expanded range of λ values, with a logarithmic scale on the *y*-axis. **(b)** The conditional variance of *T*_λ,*h*_, given by (13), is decreasing as λ increases. **(c)** The probability that the diffusing molecule reaches the nucleus, *Z*_λ,*h*_, is strictly decreasing as λ increases, illustrating Theorem 1.

In Fig 3 we consider statistics of *T*_λ,*h*_ when the diffusing molecule is initially placed randomly on the cell membrane (i.e. the uniform initial condition (5)). Fig 3a illustrates Corollary 1, showing that for each cell 〈*T*_λ,*h*_〉 is strictly decreasing as λ is increased. Similarly, Fig 3c illustrates Theorem 1, showing that the probability the molecule reaches the nucleus, *Z*_λ,*h*_, is strictly decreasing as λ increases. In Fig 3b we examine the conditional variance of *T*_λ,*h*_, defined by

$$\begin{array}{cc} \mathrm{Var}[T_{\lambda ,h}] & :=E[{\left(T_{\lambda ,h}-\left\langle T_{\lambda ,h}\right\rangle \right)}^{2}\;|\;T_{\lambda ,h}<\infty ]=\frac{\int_{0}^{\infty }\left(t^{2}-{\left\langle T_{\lambda ,h}\right\rangle }^{2}\right)e^{-\lambda t}f_{h}\left(t\right)\;dt}{\int_{0}^{\infty }e^{-\lambda t}f_{h}\left(t\right)\;dt}. \end{array}$$ (13)

In each B cell the conditional variance is strictly decreasing. In Fig E, Fig F and Fig G of S1 Text we show that similar results hold when the diffusing molecule’s initial position is more localized. There the molecule is initially placed randomly within small patches of the cell membrane, see Section SI2 of S1 Text for details.

### Inactivation can sharpen the signal reaching the nuclear membrane

To understand how inactivation can affect signal propagation, we investigate how the signal reaching the nucleus changes as the inactivation rate λ is increased, but the number of molecules reaching the nucleus is held fixed. By fixing the number of molecules (i.e. total signal) that ultimately reach the nucleus, we can investigate how inactivation influences signal timing without modulating the total signal strength. Note, to fix the total signal reaching the nucleus requires that an increasing number of signaling molecules be released from the cell membrane as λ increases.

Consider a deterministic version of (10). Assume *N*_0_ molecules are initially uniformly distributed across the interior of the cell membrane, and let *u*_*h*_(***x***_*i*_, *t*) denote the (deterministic) concentration of molecules located at ***x***_*i*_ at time *t*. We assume *u*_*h*_ has units of number per (*μ*m)^3^. *u*_*h*_ then also satisfies (10), but with the initial condition

$$u_{h}\left(x_{i},0\right)=N_{0}g_{h}\left(x_{i}\right),\;V_{i}\in C_{h},$$

so that *u*_*h*_(***x***_*i*_, *t*) = *N*_0_ *p*_λ,*h*_(***x***_*i*_, *t*). The number of molecules per time that successfully reach the nucleus is given by the total flux of *u*_*h*_ into the nucleus, *N*_0_ *f*_λ,*h*_(*t*). Similarly, the total number of molecules to successfully reach the nucleus is

$$N=N_{0}\int_{0}^{\infty }f_{\lambda ,h}\left(t\right)\;dt=N_{0}Z_{\lambda ,h}.$$

We define the signal reaching the nucleus to be the number of molecules per time that reach the nucleus, given that we assume *N* molecules overall arrive. *N*_0_ is therefore chosen so as to keep *N* fixed as the inactivation rate is varied, so that

$$N_{0}=\frac{N}{Z_{\lambda ,h}}.$$

With this choice, the signal, i.e. number of molecules per time, reaching the nuclear membrane is then $Nf_{\lambda ,h}(t)Z_{\lambda ,h}^{-1}$.

**Fig 4:**
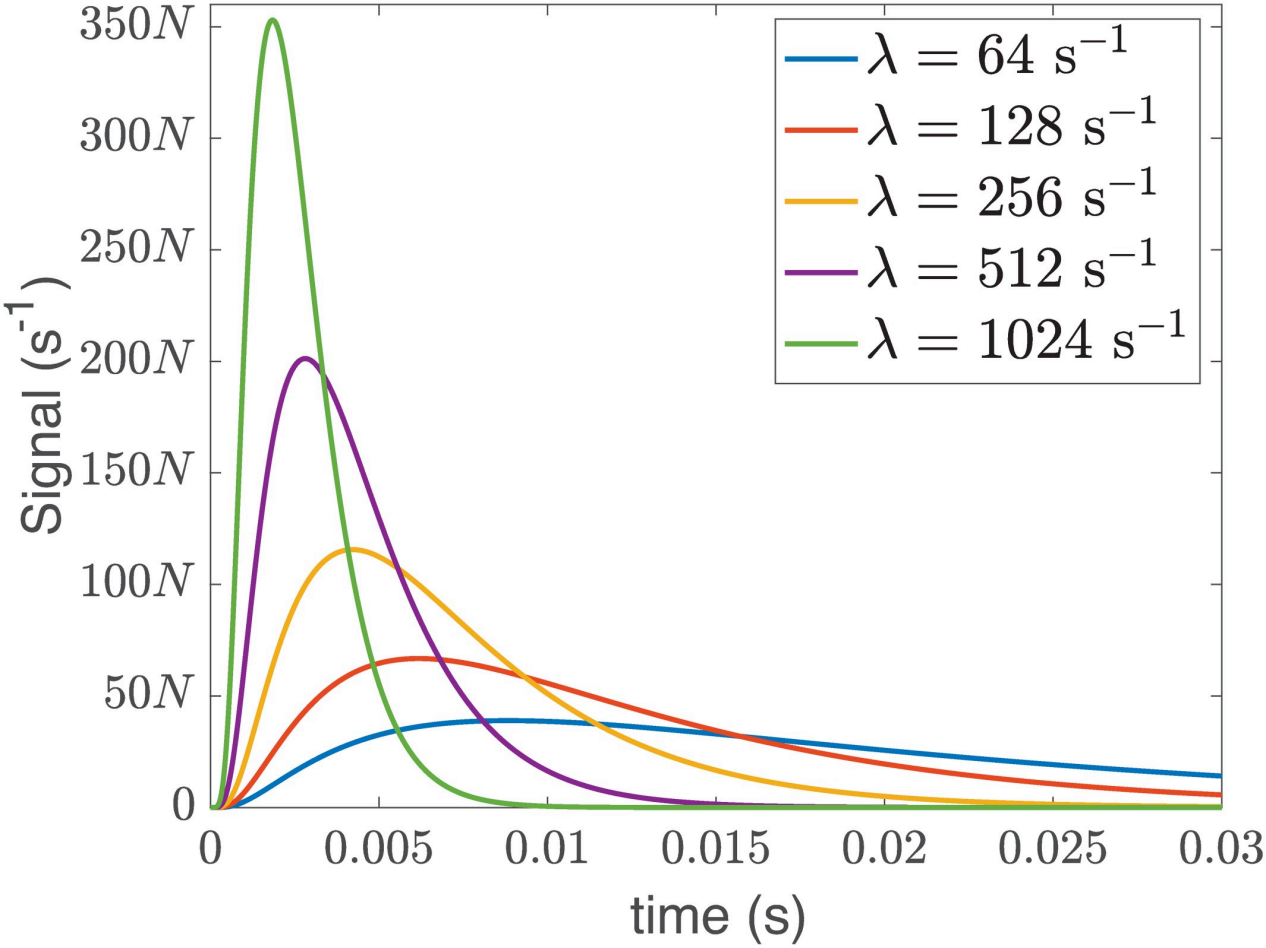
The signal in Bcell1 that successfully reaches the nuclear membrane is sharpened as the inactivation rate, λ, is increased. Here signal denotes the expected rate of arrival of signaling molecules at the nuclear membrane when the number of arriving molecules overall is *N*. The expected rate of arrival is plotted as a function of the time that has elapsed since the signaling molecules were released uniformly distributed across the interior of the cell membrane. Note that the total number of *arriving* molecules is being held constant in the results plotted here, and this requires that more signaling molecules be released when λ is greater. This is achieved by choosing the total number of molecules that are released initially as $N_{0}=NZ_{\lambda ,h}^{-1}$. As explained above, in a deterministic model with this initial condition, the signal corresponds to the flux (number of molecules per time) successfully reaching the nucleus (given by $Nf_{\lambda ,h}(t)Z_{\lambda ,h}^{-1}$). For the single-particle stochastic model (10), we can alternatively define the signal to be $f_{\lambda ,h}(t)Z_{\lambda ,h}^{-1}$. This corresponds to the single particle’s first passage time density to reach the nucleus, conditional on the molecule arriving before inactivation. The graph of this function is mathematically equivalent to the preceding figure with the units *N* = 1 on the *y*-axis. A similar signal sharpening effect is observed in Bcell2 and Bcell3, see Fig H of S1 Text.

In Fig 4 we plot the signal reaching the nucleus in Bcell1 as the inactivation rate is increased. Fig H of S1 Text shows the corresponding signals reaching the nucleus in Bcell2 and Bcell3. We see a clear sharpening effect as λ increases, with molecules arriving within an earlier and more localized time window. In this context we can interpret increasing inactivation as speeding up the arrival of the signal at the nuclear membrane. We note that in the single particle stochastic model (10), $f_{\lambda ,h}(t)Z_{\lambda ,h}^{-1}$ corresponds to the particle’s first passage time density to reach the nucleus, conditional on it reaching the nucleus before inactivation. Fig 4 therefore illustrates that the (conditional) density of random arrival times for an individual particle also undergoes sharpening as the strength of inactivation is increased (setting *N* = 1 on the *y*-axis).

While the deterministic model shows the window in which the molecules arrive becomes smaller as inactivation increases, the single-particle stochastic model (10) allows us to see how much variation one would have in the number of molecules that successfully reach the nucleus. We again assume that *N*_0_ signaling molecules are activated uniformly on the interior of the cell membrane, and that the molecules’ dynamics are *completely independent*. The number of molecules that reach the nucleus would then be a binomial random variable, **N** ∼ *B*(*N*_0_, *Z*_λ,*h*_), in *N*_0_ with parameter *Z*_λ,*h*_. The average number of molecules to reach the nucleus would be $E\left[N\right]=N_{0}Z_{\lambda ,h}$, while the coefficient of variation in the number of molecules to reach the nucleus is

$$\begin{array}{c} \mathrm{CV}[N]=\sqrt{\frac{1-Z_{\lambda ,h}}{N_{0}Z_{\lambda ,h}}}\approx \frac{1}{\sqrt{E[N]}} \end{array}$$ (14)

for λ large. Here we have used that the probability to reach the nucleus, *Z*_λ,*h*_ approaches zero as λ → ∞, see the next section, and approximated the square root in the numerator by the leading-order term of its Taylor series expansion about *Z*_λ,*h*_ = 0. Keeping *N*_0_*Z*_λ,*h*_ fixed as the inactivation rate is increased then preserves the expected number of molecules to reach the nucleus. Moreover, (14) demonstrates that the relative variation in the number of molecules that reach the nucleus will be small if the average number of molecules that reach the nucleus, E[N], is sufficiently large. By modulating both the inactivation rate and the number of signaling molecules released at the cell membrane, a cell can then tune both how localized the signal is in time, and the noisiness in the number of molecules that successfully reach the nuclear membrane.

### Inactivation can provide robustness with respect to cellular substructure in the time for a signal to reach the nucleus

**Fig 5:**
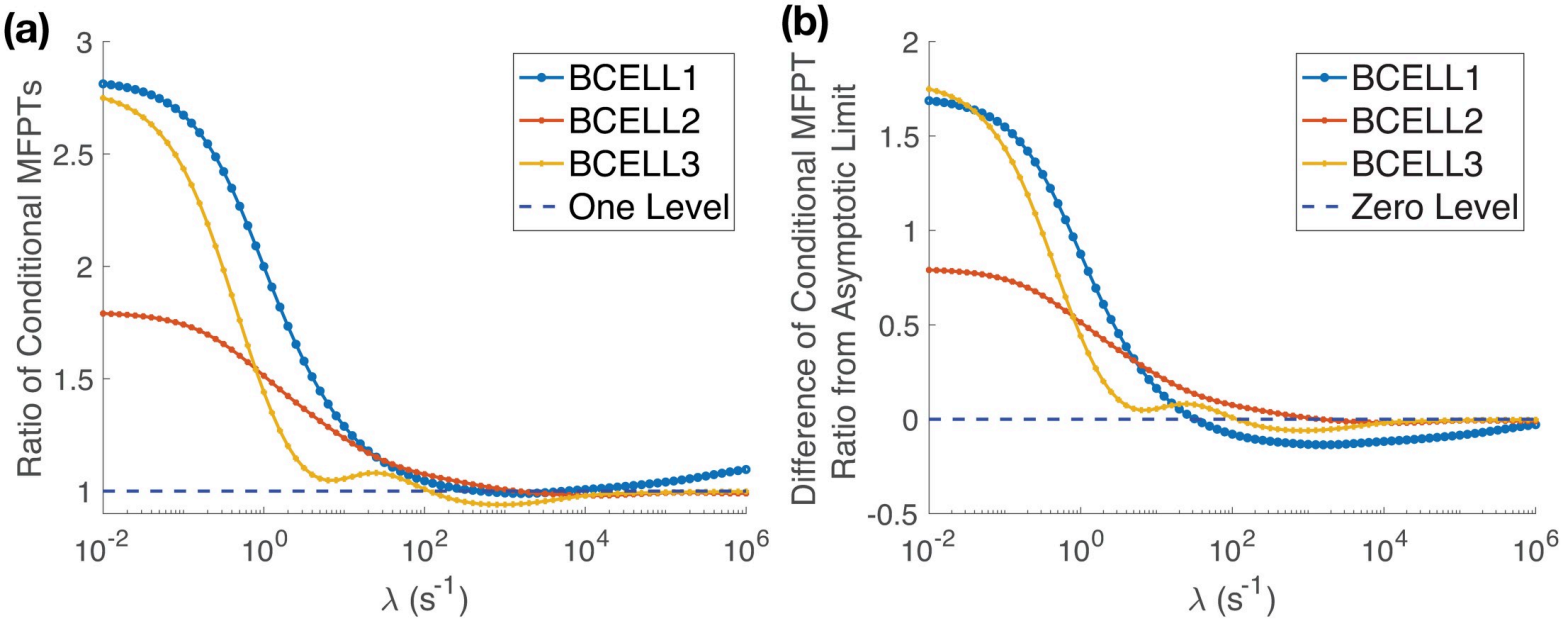
Strong signal inactivation can buffer out the effects of cellular substructure on the time to find the nucleus. **(a)** The ratio of the conditional mean first passage time (MFPT) to reach the nucleus, 〈*T*_λ,*h*_〉, in the physiological case to the conditional MFPT in the no organelles case decreases significantly from its initial value as λ increases. For each cell the ratio approaches a number close to one, indicating that strong signal inactivation can completely buffer out the effect of cellular substructure on the time to find the nucleus. **(b)** Difference of the ratio of 〈*T*_λ,*h*_〉 shown in (a) from its asymptotic limit (18). Note, (b) demonstrates that the slight increase above one for the ratio (18) in Bcell1 is just the approach to its asymptotic limit, 1.125. The ratios (18) for Bcell2 and Bcell3 both converge to 1 in (a).

In Fig 5a we plot the ratio of 〈*T*_λ,*h*_〉 in the physiological case to the no organelles case. For very small values of the inactivation rate the figure demonstrates that the presence of organelles can significantly increase the time required for one diffusing molecule to reach the nucleus. In contrast, as λ increases, for each B cell we see that the ratio decreases to a value close to one. That is, strong signal inactivation seems to be able to buffer out the effect of cellular geometry. This comes at the cost of a significantly decreased probability any individual signaling molecule will reach the nucleus.

These simulations illustrate that the ratio of the MFPTs between the physiological and no organelle cases is decreased for sufficiently strong signal inactivation. To understand the limit to how much strong signal inactivation can buffer out the effect of organelle barriers in our model, we now examine the large λ asymptotic expansion of the conditional MFPT, 〈*T*_λ,*h*_〉. Our goal is to derive an explicit formula for the asymptotic limit of 〈*T*_λ,*h*_〉 as λ → ∞ that illustrates the role of the geometry of the cytosolic space. Our derivation demonstrates how the effect of geometry on the limiting conditional MFPT arises. Readers interested solely in the derived formula may skip ahead to (17).

By (12), knowing the asymptotic behavior of *Z*_λ,*h*_ as λ → ∞ would allow us to calculate the behavior of 〈*T*_λ,*h*_〉. In turn, the behavior of *Z*_λ,*h*_ can be calculated from the integral representation (11). This will be determined by the short-time behavior of *f*_*h*_(*t*) due to the rapid decay of the exponential for large λ. We therefore begin by examining the behavior of *f*_*h*_ as *t* → 0. We can estimate this short-time behavior by direct Taylor series expansion using a matrix exponential representation for the evolution operator, i.e.

$$\begin{array}{cc} f_{h}\left(t\right) & =-\frac{dS_{h}}{dt}=-\sum_{V_{i}\in C_{h}}\frac{dph}{dt}\left(x_{i},t\right)h^{3}\\ & =-Dh^{3}\sum_{V_{i}\in C_{h}}(\Delta_{h}e^{D\Delta_{h}t}g_{h})\left(x_{i},t\right)\\ & =-h^{3}\sum_{V_{i}\in C_{h}}\sum_{n=0}^{\infty }[\frac{{\left(D\Delta_{h}\right)}^{n+1}t^{n}}{n!}g_{h}]\left(x_{i}\right)\\ & =-h^{3}\sum_{n=0}^{\infty }\sum_{V_{i}\in C_{h}}\frac{D^{n+1}t^{n}}{n!}({\left(\Delta_{h}\right)}^{n+1}g_{h})\left(x_{i}\right). \end{array}$$ (15)

To simplify this expression we make use of the relationship between powers of the discrete Laplacian and geodesic (nearest-neighbor) graph distances.

Recall our assumption that *g*_*h*_(***x***_*i*_) = 0 for all ***x***_*i*_ ∉ ∂*C*_*h*_, and denote by $G_{h}\subset \partial C_{h}$ the set of voxels in which *g*_*h*_(***x***_*i*_) ≠ 0 (i.e. the support of *g*_*h*_). If the particle is started randomly placed within the voxels bordering the cell membrane then $G_{h}=\partial C_{h}$, whereas if the particle is initially started at a fixed point, ***x***_*i*_, then $G_{h}=\{x_{i}\}$. Given a set of voxels $V\subset C_{h}$, we define $d(V,N_{h})$ to be the shortest (integer) graph distance along a nearest-neighbor path from each voxel in V to first reach a voxel in *N*_*h*_. Here by nearest-neighbor we mean the six nearest-neighbors to a given voxel, two from each of the ***x***, ***y*** and ***z*** directions. For example, if no voxel in V is within *N*_*h*_, but *some* voxel in V has a nearest neighbor that is within *N*_*h*_, then $d(V,N_{h})=1$.

It is from the powers of the discrete Laplacian in (15) that the role of cytosolic geometry in the short-time behavior of *f*_*h*_(*t*) arises, ultimately dictating the large λ behavior of 〈*T*_λ,*h*_〉. As shown in Lemma 1 of S1 Text, the {*V*_*i*_ ∈ *C*_*h*_|(Δ_*h*_)^*k*^ *g*_*h*_(***x***_*i*_) ≠ 0} will contain no voxels *bordering* the nucleus until $k=d(G_{h},N_{h})-1$. For any smaller *k*, one additional application of the discrete Laplacian then simply moves probability mass within the cytosol. As such, mass is conserved and we have the following result which is proven in Section SI1 of S1 Text

**Theorem 2**

$$\sum_{V_{i}\in C_{h}}({\left(\Delta_{h}\right)}^{k}g_{h})\left(x_{i}\right)=0$$

*for* $1\leq k\leq d(G_{h},N_{h})-1$.

With $d_{g}=d(G_{h},N_{h})$, the theorem implies that (15) can be simplified to

$$\begin{array}{cc} f_{h}\left(t\right) & =-h^{3}\sum_{n=d_{g}-1}^{\infty }\sum_{V_{i}\in C_{h}}\frac{D^{n+1}t^{n}}{n!}({\left(\Delta_{h}\right)}^{n+1}g_{h})\left(x_{i}\right)\\ & ∼-h^{3}\frac{D^{d_{g}}t^{d_{g}-1}}{(d_{g}-1)!}\sum_{V_{i}\in C_{h}}({\left(\Delta_{h}\right)}^{d_{g}}g_{h})\left(x_{i}\right),\;\;\text{as}\;t\to 0. \end{array}$$

Assuming that *d*_*g*_ > 1, we obtain the corresponding estimate for *Z*_λ,*h*_ as λ → ∞ by

$$\begin{array}{c} \begin{array}{cc} Z_{\lambda ,h} & =\int_{0}^{\infty }e^{-\lambda t}f_{h}\left(t\right)\;dt=\frac{1}{\lambda }\int_{0}^{\infty }e^{-s}f_{h}\left(s\lambda^{-1}\right)\;ds\\ & ∼-h^{3}\frac{D^{d_{g}}}{\lambda^{d_{g}}}\sum_{V_{i}\in C_{h}}({\left(\Delta_{h}\right)}^{d_{g}}g_{h})\left(x_{i}\right),\;\;\text{as}\;\lambda \to \infty . \end{array} \end{array}$$ (16)

In Theorem 3 of S1 Text we prove this asymptotic formula holds. Taking logarithmic derivatives, we find that

$$\begin{array}{c} \left\langle T_{\lambda ,h}\right\rangle =-\frac{d}{d\lambda }\log\left(Z_{\lambda ,h}\right)∼\frac{d(G_{h},N_{h})}{\lambda },\;\;\text{as}\;\lambda \to \infty . \end{array}$$ (17)

In Fig D of S1 Text we show the convergence of 〈*T*_λ,*h*_〉 to this asymptotic formula as λ → ∞.

Let $d(G_{h},N_{h}{)}_{\text{phys}}$ denote the distance from $G_{h}$ to the nucleus in the physiological case, with $d{\left(G_{h},N_{h}\right)}_{\text{n}.\text{o}.}$ the distance in the no organelle case. Define 〈*T*_λ,*h*_〉_phys_ and 〈*T*_λ,*h*_〉_n.o._ analogously. The ratio of the conditional MFPTs then approaches

$$\begin{array}{c} \frac{{\left\langle T_{\lambda ,h}\right\rangle }_{\text{phys}}}{{\left\langle T_{\lambda ,h}\right\rangle }_{\text{n}.\text{o}.}}∼\frac{d{\left(G_{h},N_{h}\right)}_{\text{phys}}}{d{\left(G_{h},N_{h}\right)}_{\text{n}.\text{o}.}},\;\text{as}\;\lambda \to \infty . \end{array}$$ (18)

That is, how much the effect of geometry on the search time can be buffered out by strong inactivation in our model is essentially controlled by how the shortest path (nearest-neighbor) graph distance from the initial set the particle can be placed in to the nucleus changes between the physiological and no organelle cases. In particular, since the voxels within the cytosol in the physiological case are always a strict subset of those in the no organelles case, we see the ratio is always at least one (in the limit).

In Fig 5b we plot the difference between the ratio of the conditional MFPTs and the derived asymptotic limit in (18). We see that for each cell the asymptotic limit is approached as λ → ∞, but that the approach is not always monotonic. In particular, the asymptotic limit (18) does not appear to be a rigorous lower bound for how much the effect of geometry can be buffered out over all possible inactivation rates.

If the diffusing molecule is started at a fixed location, ***x***_*i*_, we obtain

$$\frac{{\left\langle T_{\lambda ,h}\right\rangle }_{\text{phys}}}{{\left\langle T_{\lambda ,h}\right\rangle }_{\text{n}.\text{o}.}}∼\frac{d{\left(x_{i},N_{h}\right)}_{\text{phys}}}{d{\left(x_{i},N_{h}\right)}_{\text{n}.\text{o}.}},\;\text{as}\;\lambda \to \infty ,$$

the ratio of the shortest graph (nearest-neighbor) distances from ***x***_*i*_ to the nucleus in the two cases. In particular, if the shortest path distance from ***x***_*i*_ to the nucleus is the same in both cases, we find that the effect of organelle barriers on the conditional MFPT is completely filtered out in the limit of strong signal inactivation.

In Section SI2 of S1 Text, we show analogous results to Fig 5 when the diffusing molecule is started randomly within small patches of the cell membrane. We see similar qualitative behavior for statistics of *T*_λ,*h*_, and for the ratio of 〈*T*_λ,*h*_〉 in the physiological to no organelles cases. Note, however, that we observe a variation in how much the effect of geometry can be buffered out as the patch of cell membrane where the signal is initiated moves about.

## Discussion

Our results demonstrate that organelle barriers to the molecular diffusion of signaling molecules can significantly slow the propagation of a signal from the cell membrane to the nucleus. Such barriers also increase the variability in the distribution of times to reach the nucleus for signals activated at different localized portions of the cell membrane. Strong signal inactivation provides one potential mechanism to both buffer out the effect of organelle barriers, and to reduce variability in the time at which signals reach the nucleus. Mechanisms to reduce such variability may be needed to ensure robust functioning of pathways that involve pulsatile responses. For example, the relative expression of the pituitary hormones LH and FSH is controlled by the pulse frequency of extracellular GnRH ligands [21]. Sufficient variability in processing such signals might lead to improper expression levels through misidentification of the pulse frequency.

Under the constraint that the expected number of molecules to reach the nucleus should be *fixed* at *N*, the inactivation rate can be adjusted provided that the initial number of molecules activated at the inner surface of the cell membrane are varied in a compensating manner. Under these assumptions, Fig 4 demonstrates that the time for a signal to reach the nuclear membrane can be made arbitrarily small by increasing the inactivation rate. This comes with a clear cost though; increasing the rate of signal inactivation requires increasing numbers of signaling molecules to be activated at the cell membrane to maintain a fixed number of molecules that successfully reach the nucleus.

Our conclusions can be generalized in several ways. First, while we focused on the propagation of a signal between the cell and nuclear membranes, our results should hold more generally for a variety of signal sources and targets within cells. In more general signaling pathways they should also apply to the most downstream signaling component, presuming it is not activated right near the nuclear membrane. Finally, we note that while signaling pathways can involve complicated reaction kinetics throughout the cytosol, it may be that in some cases their overall effect can be approximated as a single signal that propagates throughout the cytosol and is inactivated on some timescale.

**Regime of Model Applicability:** It is important to note that the large λ asymptotic scaling in (17), and convergence to the ratio (18), may require relatively large values of λ (on the order of λ between 10^4^ s^−1^ and 10^6^ s^−1^ for *D* = 10(*μ*m)^2^s^−1^, see Fig 5b and Fig D of S1 Text). Molecules that successfully reach the nucleus would on average arrive on time scales of 10^−4^s^−1^ or less, see Fig D of S1 Text, which would not necessarily be expected to be physically plausible in a typical mammalian cell. More generally, as λ → ∞ these results rely on the (increasingly) short-time behavior of the continuous-time random walk model (10). However, both the continuous diffusion model (7) and the continuous time random walk model (10) become physically unrealistic as models for the very short-time motion of a molecule within a cell. Moreover, the *very* short-time behavior of the semi-discrete model (10) and the continuous diffusion model (7) would not be expected to agree, since the former only approximates the latter on sufficiently large timescales.

The relative behavior of the two models is illustrated in Fig I and Section SI3 of S1 Text. There we compare the analytical PDE solution, when the nuclear membrane and cell membrane are represented as concentric spheres, to the numerical solution of the corresponding semi-discrete model on a Cartesian grid approximation of the cytosolic region between the spheres. We find that for a mesh spacing of *h* = 0.0351*μ*m, comparable to that of our B cell reconstructions, 〈*T*_λ_〉 and 〈*T*_λ,*h*_〉 agree exceptionally well until the asymptotic λ^−1^ scaling takes over in the semi-discrete model. Then we see a discrepancy due to the different short-time behavior of the semi-discrete model, with the λ^−1^ scaling, and the exact solution to the continuous diffusion PDE, which exhibits a λ^−1/2^ scaling, see (SI5) in S1 Text.

For these reasons the usefulness of understanding the large λ asymptotic behavior is not in the predicted scaling of 〈*T*_λ,*h*_〉 (17), but in the decreasing asymptotic behavior of the conditional MFPT ratio (18). This asymptotic limit provides insight into why, on physiological timescales, we observe a decrease in the effect of organelle barriers on signal propagation. Namely, signal inactivation filters out the molecules that would have had to traverse longer paths to get to the nucleus. This reduces differences between the lengths of paths which molecules that reach the nucleus must take in the organelle filled, and organelle empty, cell.

**Conjectures and Open Problems:** For the continuous diffusion model (7), let G denote the set on which *g*(*x*) ≠ 0 (i.e. the support of *g*(*x*)). For example, if the particle is started uniformly on the inner surface of the cell membrane than G=∂C. We conjecture that the corresponding ratio of conditional MFPTs satisfies

$$\frac{{\left\langle T_{\lambda }\right\rangle }_{\text{phys}}}{{\left\langle T_{\lambda }\right\rangle }_{\text{n}.\text{o}.}}∼\frac{d{\left(G,\partial N\right)}_{\text{phys}}}{d{\left(G,\partial N\right)}_{\text{n}.\text{o}.}},\;\text{as}\;\lambda \to \infty ,$$

where d(G,∂N) refers to the shortest path geodesic distance through the cytosol from the signal initiation location, G, to the nuclear membrane ∂*N*. We have obtained partial results to this effect when there are straight line paths from G to ∂*N* and the principal curvatures of the nuclear membrane satisfy certain constraints, but the general case remains an open problem.

The geodesic distance has recently been suggested to also arise in the context of the first searcher problem. Here one is interested in the average time at which the first of *N* searchers reaches a target as the number of searchers, *N*, becomes large (i.e. *N* → ∞). In [22] it was suggested that, similar to our observations for strong signal inactivation, this limit also filters out all but the shortest paths, with the average time for the first searcher to reach a target scaling like the square of the geodesic distance. An interesting future question would be to understand the interplay of these two problems; i.e. the time required for the first of many searchers to successfully reach a binding target in the presence of strong signal inactivation.

Finally, we note that it is an open question to understand whether spatial signaling pathways [3, 23, 24] involve more general mechanisms for filtering out the effect of spatial heterogeneity within the cytosolic environment. It would be particularly interesting to investigate such questions while also studying the role of two effects that we have not explicitly resolved; crowding between molecules within the cytosol and active transport of signaling molecules to the nuclear membrane. In addition, in this work we considered only the simplest of signaling components: linear inactivation. For many signaling pathways, including BCR signaling in B cells and general protein kinase signaling, inactivation is more appropriately modeled as occurring through a nonlinear interaction with a phosphatase [4, 5]. Such pathways also commonly involve cascades of interactions [3], which could conceivably have additional mechanisms that buffer out the influence of cellular substructure on signal timing. We hope to explore such models in future work.

Cell signaling and computational modeling are an enormous field with a breadth of studies, both spatial and non-spatial, that have been carried out, see the many references of [25, 26]. Within the field a variety of studies have investigated the spatial dynamics of cell signaling, which can be critical to the proper function and decision making of cells, see the review [23] and references. In particular, one focus within these works is understanding how cell shape and organelle positioning can influence signaling [1, 4], the former reviewed in [27]. Our work complements such studies, demonstrating how internal organelle barriers can impact signaling, and provides insight into mechanisms that regulate the timing of signal propagation. It represents another step in developing detailed, anatomically accurate whole-cell spatial models that can account for the inherent stochasticity in both spatial transport and chemical reactions [28].

## Methods

### Reconstruction of cellular substructure

To reconstruct the locations of organelles and membrane surfaces, we made use of soft X-ray tomographic (SXT) imaging of cells. For an overview of SXT imaging, we refer the reader to [11]. In this work we used reconstructions of three human B cells (GM12878 lymphoblastoids) from [29]. The experimental protocol for obtaining these reconstructions was also described in [29]. SXT is similar in concept to medical X-ray CT imaging, but uses soft X-rays in the “water window,” which are absorbed by carbon and nitrogen dense organic matter an order of magnitude more strongly than by water [11]. As the absorption process satisfies the Beer—Lambert law, the measured linear absorption coefficient (LAC) of one voxel of a 3D reconstruction is linearly related to the density of organic material within that voxel [11]. In practice, SXT reconstructions are able to achieve resolutions of 50 nm or less. For all reconstructions used in this work, the underlying voxels were cubes with sides of length 0.03515625*μ*m. Another advantage of SXT is in the minimal preprocessing of cells that is required before imaging. Cells are cryogenically preserved, but no segmentation, dehydration, or chemical fixation is necessary. Fig 1a shows the reconstructed LAC values from one image plane within a 3D SXT reconstruction of Bcell1.

As discussed in [30], many organelles have different underlying densities of organic material, and therefore attenuate soft X-rays differently. This is reflected in their having different LAC values. Exploiting this property, 3D SXT reconstructions were labeled and segmented in Amira [31], using a combination of Amira’s automated segmentation tools based on LAC values, followed by hand segmentation to refine segmentation boundaries [30]. Each underlying voxel within the 3D SXT reconstruction was labeled as belonging to one of a variety of organelles (heterochromatin, euchromatin, endoplasmic reticulum, mitochondria, Golgi apparatus, bulk cytosol, etc.). Fig 1c shows one plane of the resulting label field.

### Numerical solution of semi-discrete diffusion eq (3)

The semi-discrete diffusion eq (3) was solved in PETSc 3.7.7 [32, 33] using the adaptive Runge-Kutta Chebyshev (RKC) method of [34] with both the absolute and relative errors set to 10^−8^. To evaluate the solution, *p*_*h*_(***x***, *t*), at larger times, it was approximated by a truncated eigenvector expansion using all terms with eigenvalues having a magnitude less than one. The corresponding eigenvalues and eigenvectors of the discrete Laplacian (4) were calculated in SLEPc 3.7.4 [35] using the Krylov-Schur solver with default parameter values and tolerances. For all simulations the decision to switch from the RKC solver to the eigenvector expansion was made by looking over the interval 1 < *t* < 10 for where the two solutions first differed by an absolute error of less than 10^−5^ and a relative error of less than .01.

To numerically evaluate the integrals defining statistics such as *Z*_λ,*h*_ and 〈*T*_λ,*h*_〉, we split them into two pieces. The integral from zero to the time at which the PDE solver switched from the RKC method to the truncated eigenvector expansion, and the integral from this time to infinity. The first integral was evaluated using the cumulative trapezoidal rule at the discretization times used in the RKC method. The second integral was evaluated by analytically integrating the truncated eigenvector expansion. Within these integrals the probability density function for the molecule to reach the nucleus was calculated directly from the flux into voxels of the nucleus,

$$f_{h}\left(t\right)=\frac{D}{h^{2}}\sum_{i=1}^{M}\sum_{j\in N(V_{i};N_{h})}p_{h}\left(x_{i},t\right),$$

using the numerically computed solutions.

## Supporting information

S1 Text Supporting information for “Strong intracellular signal inactivation produces sharper and more robust signaling from cell membrane to nucleus”. (PDF)
