# Supplementary material for: Strong intracellular signal inactivation produces sharper and more robust signaling from cell membrane to nucleus
Source: PLoS Comput Biol. 2020 Nov 16;16(11):e1008356. doi: 10.1371/journal.pcbi.1008356 (PMC7704053; doi:10.1371/journal.pcbi.1008356)
Supplement: S1 Text — (PDF) [file pcbi.1008356.s001.pdf]

SUPPORTING INFORMATION FOR "STRONG INTRACELLULAR SIGNAL INACTIVATION PRODUCES SHARPER AND MORE ROBUST SIGNALING FROM CELL MEMBRANE TO NUCLEUS"

SI1. Proofs of Main Text Theorems

In this section we give proofs of Theorem 1, Corollary 1 and Theorem 2. We begin with Theorem 1 and Corollary 1. In our proofs, we do not show here, but make use of, the following basic results on properties of the solutions to (1) and (7).

1. The probability per time the diffusing molecule reaches the nucleus in the model without degradation, $f(t)$, is positive for $t > 0$.
2. The conditional survival probability $S_\lambda(t) = 1 - F_\lambda(t)$ approaches zero as $t \to \infty$ sufficiently fast that $tS_\lambda(t) \to 0$ and $S_\lambda(t)$ is integrable.

Theorem 1. *For all fixed $t > 0$ and $\lambda \geq 0$, $Z_\lambda(t)$ is a strictly decreasing function of λ, and $F_\lambda(t)$ is a strictly increasing function of λ.*

Proof. Let $\lambda_2 > \lambda_1 \geq 0$, it is immediate that $Z_{\lambda_1} > Z_{\lambda_2}$. Showing $F_{\lambda_2}(t) > F_{\lambda_1}(t)$ for all $t > 0$ is equivalent to showing

$$H(t) = Z_{\lambda_1} \int_0^t f_{\lambda_2}(s)\, ds - Z_{\lambda_2} \int_0^t f_{\lambda_1}(s)\, ds > 0$$

for $t > 0$. Note $H(0) = 0$ and $\lim_{t\to\infty} H(t) = 0$. We then have that

$$H'(t) = Z_{\lambda_1} f_{\lambda_2}(t) - Z_{\lambda_2} f_{\lambda_1}(t) = f(t) \left(Z_{\lambda_1} e^{-\lambda_2 t} - Z_{\lambda_2} e^{-\lambda_1 t} \right).$$

As such, using that $f(t) > 0$ for $t > 0$, we have that $H'(t) = 0$ if and only if

$$\frac{Z_{\lambda_1}}{Z_{\lambda_2}} = e^{(\lambda_2 - \lambda_1)t}.$$

As the left side is greater than one, there is exactly one point, t^*, where equality can hold for $0 < t < \infty$. For $0 < t < t^*$, $H'(t) > 0$, while for $t > t^*$, $H'(t) < 0$. As such, we conclude that $H(t)$ increases from $H(0) = 0$ to a global max, and then decreases to zero as $t \to \infty$, implying that $H(t) > 0$ for $0 < t < \infty$. □

Corollary 1. *Both the conditional MFPT, $\langle T_\lambda \rangle := \mathbb{E}[T_\lambda \mid T_\lambda < \infty]$, and the conditional median first passage time, $M(T_\lambda) := F_\lambda^{-1}\left(\frac{1}{2}\right)$, are strictly decreasing with respect to λ.*

Proof. By definition

$$\langle T_\lambda \rangle = \frac{\int_0^\infty t f_\lambda(t)\, dt}{\int_0^\infty f_\lambda(t)\, dt} = -\int_0^\infty t \frac{d}{dt} \left[1 - F_\lambda(t)\right] dt,$$

where the conditional CDF, $F_\lambda(t)$, is given by (9). Note that $F_\lambda(t) \in [0, 1]$, $F_\lambda(0) = 0$ and $\lim_{t\to\infty} F_\lambda(t) = 1$. Using the assumed integrability of $tS_\lambda(t) = t(1 - F_\lambda(t))$, and integrating by parts, then gives

$$\langle T_\lambda \rangle = \int_0^\infty S_\lambda(t)\, dt = \int_0^\infty (1 - F_\lambda(t))\, dt.$$

As $F_\lambda(t)$ is strictly increasing with respect to λ for $t > 0$ and $F_\lambda(t) > 0$ for $t > 0$, this implies that $\langle T_\lambda \rangle$ is strictly decreasing in λ.

To prove the monotonicity of the median, let $\lambda_2 > \lambda_1 \geq 0$. Then

$$F_{\lambda_1}(M(\lambda_1)) = \tfrac{1}{2} = F_{\lambda_2}(M(\lambda_2)) > F_{\lambda_1}(M(\lambda_2))$$

by the monotonicity of $F_\lambda(t)$ with respect to λ. Since the CDF $F_\lambda(t)$ is non-decreasing and continuous in t, we conclude that $M(\lambda_1) > M(\lambda_2)$. □

We now prove Theorem 2 from the main text. Let $\operatorname{supp}\{w_h\}$ denote the set of voxels in C_h where a lattice function $w_h(\boldsymbol{x}_i)$ is non-zero. We first prove the following lemma, which shows that $\operatorname{supp}\{(\Delta_h)^k g_h\}$ will contain no voxels *bordering* the nucleus until $k = d(\mathcal{G}_h, N_h) - 1$. For any smaller k, one additional application of the discrete Laplacian then simply moves probability mass within the cytosol. As such, mass is conserved and we have the following result

Lemma 1. *Let* $k \in \{0, 1, \dots, d(\text{supp}\{g_h\}, N_h) - 1\}$*. Then* $d\left(\text{supp}\{(\Delta_h)^k g_h\}, N_h\right) = d(g_h, N_h) - k$*.*

Proof. The case $d(\text{supp}\{g_h\}, N_h) = 1$ is immediate. Assume $d(\text{supp}\{g_h\}, N_h) \geq 2$. Let $k \leq d(\text{supp}\{g_h\}, N_h) - 2$, and assume $\Sigma_k = \text{supp}\{(\Delta_h)^k g_h\}$ is the set of nearest neighbor shortest paths of length k from $\text{supp}\{g_h\}$. By definition of the discrete Laplacian, Σ_{k+1} then contains Σ_k, and all nearest-neighbors of Σ_k within C_h. This corresponds to all nearest-neighbor shortest paths from the voxels within $\text{supp}\{g_h\}$ of integer length $\leq k+1$, so that $d(\Sigma_k, N_h) = d(\Sigma_{k+1}, N_h) + 1$. Since $d(\Sigma_0, N_h) = d(\text{supp}\{g_h\}, N_h)$ by definition, the theorem then holds by induction. □

The lemma implies that $\text{supp}\{(\Delta_h)^k g_h\}$ will contain no voxels *bordering* the nucleus until $k = d(\text{supp}\{g_h\}, N_h) - 1$. For any smaller k, one additional application of the discrete Laplacian simply moves probability mass within the cytosol, but outside of voxels that border the nucleus. This then implies Theorem 2:

Theorem 2.

$$\sum_{V_i \in C_h} \left((\Delta_h)^k g_h\right)(\boldsymbol{x}_i) = 0$$

for $1 \leq k \leq d(\mathcal{G}_h, N_h) - 1$*.*

Proof. Assume $d(\text{supp}\{g_h\}, N_h) \geq 2$. We may then write

$$\sum_{V_i \in C_h} \left((\Delta_h)^k g_h\right)(\boldsymbol{x}_i) = \sum_{V_i \in C_h} (\Delta_h \tilde{g}_h)(\boldsymbol{x}_i),$$

where $\tilde{g}_h = \Delta_h^{(k-1)} g_h$. By Lemma 1 $\tilde{g}_h(\boldsymbol{x}_i) = 0$ for all $\boldsymbol{x}_i$ that are nearest-neighbors to voxels within the nucleus. Using (4), when acting on $\tilde{g}_h$ the discrete Laplacian then simplifies to

$$\sum_{V_i \in C_h} (\Delta_h \tilde{g}_h)(\boldsymbol{x}_i) = \frac{D}{h^2}\left[\sum_{V_i} \sum_{j \in \mathcal{N}(V_i; C_h)} \tilde{g}_h(\boldsymbol{x}_j) - \sum_{V_i} |\mathcal{N}(V_i; C_h)|\, \tilde{g}_h(\boldsymbol{x}_i)\right],$$

where $|\mathcal{N}(V_i; C_h)|$ denotes the number of neighbors of voxel V_i within C_h. Reordering the first sum we have that

$$\left[\sum_{V_i} \sum_{j \in \mathcal{N}(V_i; C_h)} \tilde{g}_h(\boldsymbol{x}_j) = \sum_{V_i} |\mathcal{N}(V_i; C_h)|\, \tilde{g}_h(\boldsymbol{x}_i)\right],$$

so that

$$\sum_{V_i \in C_h} (\Delta_h \tilde{g}_h)(\boldsymbol{x}_i) = 0.$$

□

In the following theorem we prove the asymptotic behavior of $Z_{\lambda,h}$ given in (16).

Theorem 3.

$$Z_{\lambda,h} \sim -h^3 \frac{D^{d_g}}{\lambda^{d_g}} \sum_{V_i \in C_h} \left((\Delta_h)^{d_g} g_h\right)(\boldsymbol{x}_i), \qquad \text{as } \lambda \to \infty.$$

Proof. By definition

$$Z_{\lambda,h} = \int_0^\infty e^{-\lambda t} f_h(t)\, dt = \frac{1}{\lambda} \int_0^\infty e^{-s} f_h(s\lambda^{-1})\, ds.$$

Plugging in the expansion formula for $f_h(t)$,

$$f_h(t) = -h^3 \sum_{n=d_g-1}^{\infty} \sum_{V_i \in C_h} \frac{D^{n+1} t^n}{n!} \left((\Delta_h)^{n+1} g_h\right)(\boldsymbol{x}_i),$$

we have that

$$Z_{\lambda,h} = -\, h^3 \frac{D^{d_g}}{\lambda^{d_g}} \sum_{V_i \in C_h} \left((\Delta_h)^{d_g} g_h \right)(\boldsymbol{x}_i) - \frac{h^3}{\lambda} \int_0^\infty e^{-s} \sum_{n=d_g}^{\infty} \sum_{V_i \in C_h} \frac{D^{n+1} s^n}{\lambda^n n!} \left((\Delta_h)^{n+1} g_h \right)(\boldsymbol{x}_i)\, ds.$$

In the last equation, denote the second, remainder term by I. We claim $I = o\left(\frac{1}{\lambda^{d_g}}\right)$ as $\lambda \to \infty$. We have

$$|I| \leq \frac{|C_h|}{\lambda} \int_0^\infty e^{-s} \sum_{n=d_g}^{\infty} \frac{D^{n+1} s^n}{\lambda^n n!} \| \left((\Delta_h)^{n+1} g_h \right)(\boldsymbol{x}_i) \|_{\ell^2}\, ds,$$

where $|C_h|$ denotes the volume of the cytosol and $\| \cdot \|_{\ell^2}$ denotes the discrete ℓ^2 norm over C_h. Let σ_{max} label the largest singular value of the discrete Laplacian matrix, Δ_h, then

$$\begin{aligned} |I| &\leq \frac{|C_h|}{\lambda} \int_0^\infty e^{-s} \sum_{n=d_g}^{\infty} \frac{D^{n+1} s^n}{\lambda^n n!} \sigma_{max}^{n+1} \|g_h\|_{\ell^2}\, ds \\ &= |C_h|\, \|g_h\|_{\ell^2} \sum_{n=d_g}^{\infty} \frac{D^{n+1}}{\lambda^{n+1} n!} \sigma_{max}^{n+1} \int_0^\infty e^{-s} s^n\, ds \\ &= |C_h|\, \|g_h\|_{\ell^2} \sum_{n=d_g}^{\infty} \left(\frac{\sigma_{max} D}{\lambda} \right)^{n+1}. \end{aligned}$$

Assuming we take $\lambda > \sigma_{max} D$ large enough the last series is convergent and we have

$$|I| \leq |C_h|\, \|g_h\|_{l^2} \left(\frac{\sigma_{max} D}{\lambda} \right)^{d_g+1} \left(1 - \frac{\sigma_{max} D}{\lambda} \right)^{-1} = o\left(\frac{1}{\lambda^{d_g}} \right)$$

as $\lambda \to \infty$. □

SI2. Statistics of the time to reach the nucleus with localized initial conditions

To understand how localization of the initiation of signals might influence the time for a signal to reach the nucleus, we also conducted simulations using localized patch initial conditions. This corresponded to the initial condition

$$p_h(\boldsymbol{x}_i, 0) = g_h(\boldsymbol{x}_i) = \begin{cases} \frac{1}{|A|}, & V_i \in P_h, \\ 0, & \text{else}, \end{cases} \tag{SI1}$$

where P_h denotes the set of voxels within a given patch of the cell membrane and A the area of the patch.

100 patches were determined for each cell by selecting 100 seed points on the cell membrane of the "physiological" geometries (i.e. cells with all internal organelles present). A 100-bin, equally-spaced histogram for the distribution of MFPTs across the cell membrane was generated from the values of $u_h(\boldsymbol{x}_i)$, see (6). From each bin one seed location was then randomly sampled from the collection of voxels with MFPTs within that bin. About each seed point a patch was constructed by adding all nearest-neighbor voxels of the seed point that were also within the membrane. The procedure was then repeated, adding all nearest-neighbors to previously calculated neighbors. This procedure was then repeated recursively for the newly added voxels until at least 100 voxels were obtained. The final patch then formed a connected graph within the cell membrane containing all k nearest neighbors of the seed voxel for some value k. In Fig C we show the distribution of patch diameters for the 100 patches sampled for each B cell. Typical final patch sizes were between .3 and .5 μm in diameter.

In Fig E, Fig F, and Fig G we show statistics of the conditional MFPT to reach the nucleus, $T_{\lambda,h}$, for Bcell1, Bcell2 and Bcell3 respectively. In each case we see similar qualitative behavior in the statistics to what we observed for the uniform initial condition used in the main text, see Fig 3 and Fig 5.

SI3. Comparison of Semi-Discrete Model to Continuous PDE Model: Spherically Symmetrical Case

We now investigate the accuracy in approximating, and differences between, our semi-discrete model and the continuous diffusion equation model. As our cellular reconstructions are given as labeled Cartesian meshes, we do not have an underlying spatially-continuous domain representation with corresponding analytic solution to which we can compare. We therefore instead consider a simpler problem, the spherically symmetrical case. We model the cell as a 3D ball with radius R centered at the origin, and model the nucleus as a 3D concentric ball of smaller radius r. While this problem is idealized, we will solve it using comparable mesh sizes to the B cell reconstructions, allowing us to characterize how well we resolve the continuous Brownian Motion of molecules when using this resolution in spherical geometries. In the case of continuous Brownian Motion between a spherical nucleus and cell we provide a standard, but self-contained, derivation for Z_λ and $\langle T_\lambda \rangle$. We note that in the pure-diffusive case such results are well-known [1].

We first consider an initial condition starting from a point $\boldsymbol{y}$ on the cell membrane. Taking the Laplace transform of (7) and denoting $K_{\lambda,s}(\boldsymbol{x}) = \int_0^\infty p_\lambda(\boldsymbol{x},t)e^{-st}\,dt$, we obtain

$$\begin{aligned} -\delta(\boldsymbol{y}-\boldsymbol{x}) &= D\Delta K_{\lambda,s}(\boldsymbol{x}) - (\lambda+s)K_{\lambda,s}(\boldsymbol{x}), && r < |\boldsymbol{x}| < R,\\ K_{\lambda,s}(\boldsymbol{x}) &= 0, && |\boldsymbol{x}| = r,\\ \nabla K_{\lambda,s}(\boldsymbol{x})\cdot\boldsymbol{\eta}(\boldsymbol{x}) &= 0, && |\boldsymbol{x}| = R. \end{aligned} \tag{SI2}$$

Let $F_{\lambda,s}(\boldsymbol{y}) = -D\int_{\partial N} \nabla K_{\lambda,s}(\boldsymbol{x},t)\cdot\boldsymbol{\eta}(\boldsymbol{x})\,dA(\boldsymbol{x})$. $F_{\lambda,s}(\boldsymbol{y})$ can be solved from the following PDE:

Lemma 2. *$F_{\lambda,s}(\boldsymbol{x})$ is a solution to the following boundary value problem:*

$$\begin{aligned} D\Delta F_{\lambda,s}(\boldsymbol{x}) &= (\lambda+s)F_{\lambda,s}(\boldsymbol{x}), && r < |\boldsymbol{x}| < R,\\ F_{\lambda,s}(\boldsymbol{x}) &= 1, && |\boldsymbol{x}| = r,\\ \nabla F_{\lambda,s}(\boldsymbol{x})\cdot\boldsymbol{\eta}(\boldsymbol{x}) &= 0, && |\boldsymbol{x}| = R, \end{aligned} \tag{SI3}$$

where $\boldsymbol{\eta}(\boldsymbol{x})$ denotes the unit outward normal to the sphere $\partial B(0,R) = \{|\boldsymbol{x}| = R\}$.

Proof. Multiplying (SI3) by $K_{\lambda,s}(\boldsymbol{x})$ and applying Green's identity, we obtain

$$\begin{aligned} F_{\lambda,s}(\boldsymbol{y}) = D&\int_{\partial B(0,R)\cup\partial B(0,r)} K_{\lambda,s}(\boldsymbol{x})\nabla F_{\lambda,s}(\boldsymbol{x})\cdot\boldsymbol{\eta}(\boldsymbol{x})\,dA(\boldsymbol{x})\\ &- D\int_{\partial B(0,R)\cup\partial B(0,r)} F_{\lambda,s}(\boldsymbol{x})\nabla K_{\lambda,s}(\boldsymbol{x})\cdot\boldsymbol{\eta}(\boldsymbol{x})\,dA(\boldsymbol{x}). \end{aligned}$$

Plugging in the boundary conditions of (SI2) and (SI3), we find $F_{\lambda,s}(\boldsymbol{y}) = -D\int_{\partial N}\nabla K_{\lambda,s}(\boldsymbol{x},t)\cdot\boldsymbol{\eta}(\boldsymbol{x})\,dA(\boldsymbol{x})$. □

Solving (SI3), we obtain

$$F_{\lambda,s}(\boldsymbol{y}) = \frac{G_{\lambda,s}(|\boldsymbol{y}|)}{G_{\lambda,s}(r)}, \tag{SI4}$$

where

$$G_{\lambda,s}(w) = \frac{1}{w}\left((mR+1)e^{-m(R-w)} + (mR-1)e^{m(R-w)}\right)$$

and

$$m = \sqrt{\frac{\lambda+s}{D}}.$$

The probability that starting from a point $\boldsymbol{y}$, the molecule reaches the nuclear membrane before inactivation can be rewritten as

$$Z_\lambda = \int_0^\infty \left(-D\int_{\partial N}\nabla p_\lambda(\boldsymbol{x},t)\cdot\boldsymbol{\eta}(\boldsymbol{x})\,dA(\boldsymbol{x})\right)dt = F_{\lambda,0}(\boldsymbol{y}).$$

From (SI4), this probability only depends on the length of the initial position, $|\boldsymbol{y}|$. Therefore, the same exit time statistics hold for a uniformly distributed initial condition starting from the cell membrane, i.e. $|\boldsymbol{y}| = R$, so that

$$Z_\lambda = \frac{G_{\lambda,0}(R)}{G_{\lambda,0}(r)}.$$

The conditional mean first passage time can be calculated from

$$\langle T_\lambda \rangle = -\frac{d}{d\lambda} \ln(Z_\lambda).$$

In particular, when $\lambda \to 0$ we obtain

$$\langle T_0 \rangle = \frac{1}{6} \left(2 \left(\frac{R}{r} \right) + 1 \right) \left(\frac{(R-r)^2}{D} \right),$$

and when $\lambda \to \infty$,

$$\langle T_\infty \rangle \sim \frac{(R-r)}{2\sqrt{D\lambda}}. \tag{SI5}$$

As we mention in the main text Discussion, this result is reflective of the short-time behavior of the signaling molecule's continuous Brownian motion. Since random walks do not approximate Brownian motions on sufficiently short time scales, as expected the $\lambda^{-1/2}$ scaling we obtain is different than the λ^{-1} scaling we proved for the semi-discrete model.

For numerical comparison, we generated a cell-centered 3D Cartesian mesh to approximate the spherically symmetrical geometry, where if the center of a voxel is within the ball of radius $r = 3\mu$m we identified it as being in the nucleus. Likewise, if the center of a voxel is within the ball radius of $R = 5\mu$m but outside the nucleus, we identified it being in the cytosol. Voxels cut by the sphere of radius $R = 5\mu$m were identified as belonging to the cell membrane. The mesh width in our simulations was $h = 0.0351\mu$m, which is comparable to the mesh size for each of the B cells we studied. The numerical solution method described in the Methods section was used for solving the corresponding semi-discrete model with one alteration. We used a slightly coarser absolute error tolerance of 1e-4 and relative error tolerance of .01 for determining the time at which to switch from the Runge-Kutta-Chebyshev method to the truncated eigenvector expansion.

In Fig I we compare the analytical $\langle T_\lambda \rangle$ given by the logarithmic derivative of $F_{\lambda,0}(R)$ to the numerical solution of the semi-discrete model. We see that the two solutions agree exceptionally well until the large λ asymptotic behavior takes over. Both solutions still continue to decrease as λ is further increased, but with the different asymptotic scalings discussed above. We also include a second semi-discrete solution, for a smaller mesh width of $h = 0.0175\mu$m, which used only the RKC method for solving in time. (We found it challenging to calculate the needed eigenvectors on the finer mesh, and examination of the semi-discrete solution in the coarser mesh case showed that the contribution of the eigenvector expansion at longer times to Fig I was minimal. This makes intuitive sense since the short-time behavior dominates in calculating $\langle T_{\lambda,h} \rangle$.) We see that the finer mesh results in a larger range of λ over which $\langle T_{\lambda,h} \rangle$ approximates $\langle T_\lambda \rangle$ well before the asymptotic behavior takes over.

SI4. Supplemental Figures and Tables

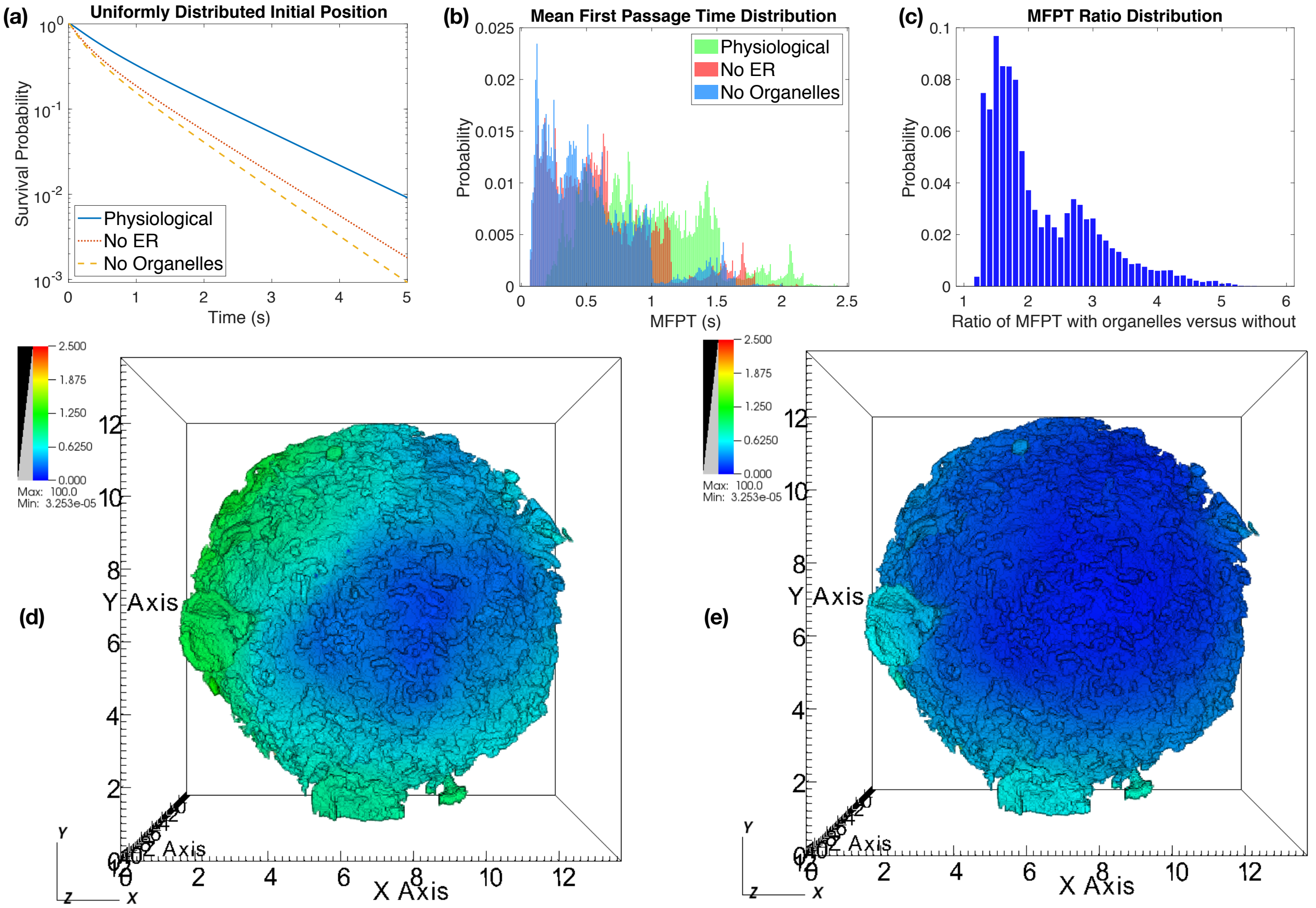

FIG. A: Statistics of MFPT in the absence of signal degradation for Bcell2. See Figure 2 for subfigure information.

	**Physiological**	**No ER**	**No Organelles**
Bcell1 Mean	0.7070	0.2721	0.2499
Bcell2 Mean	0.9540	0.6041	0.5311
Bcell3 Mean	1.6360	0.6841	0.5861
Bcell1 Median	0.4054	0.1393	0.1335
Bcell2 Median	0.5964	0.3358	0.2941
Bcell3 Median	0.8842	0.3087	0.2706
Bcell1 Variance	0.7178	0.1268	0.1007
Bcell2 Variance	1.0850	0.5390	0.4210
Bcell3 Variance	4.3352	0.9015	0.6337
Bcell1 CV	1.1983	1.3086	1.2695
Bcell2 CV	1.0919	1.2154	1.2218
Bcell3 CV	1.2726	1.3880	1.3582

TABLE A: Statistics of T_h, the random time to reach the nucleus in the absence of signal degradation in Bcell1, Bcell2 and Bcell3. The diffusing molecule is assumed to initially be randomly distributed on the cell membrane, ∂C_h. Here STD denotes standard deviation and CV denotes the coefficient of variation (the standard deviation divided by the mean).

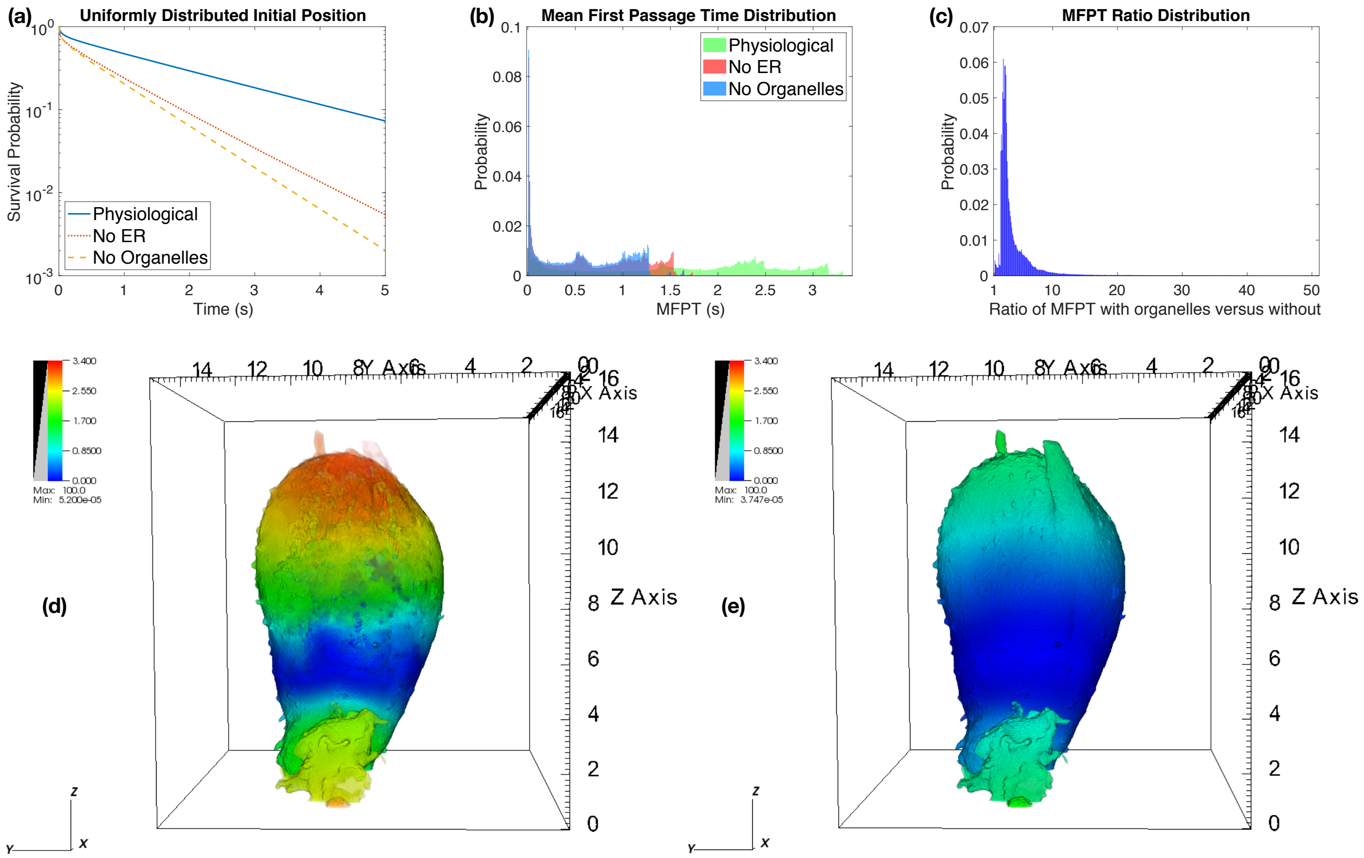

FIG. B: Statistics of MFPT in the absence of signal degradation for Bcell3. See Figure 2 for subfigure information.

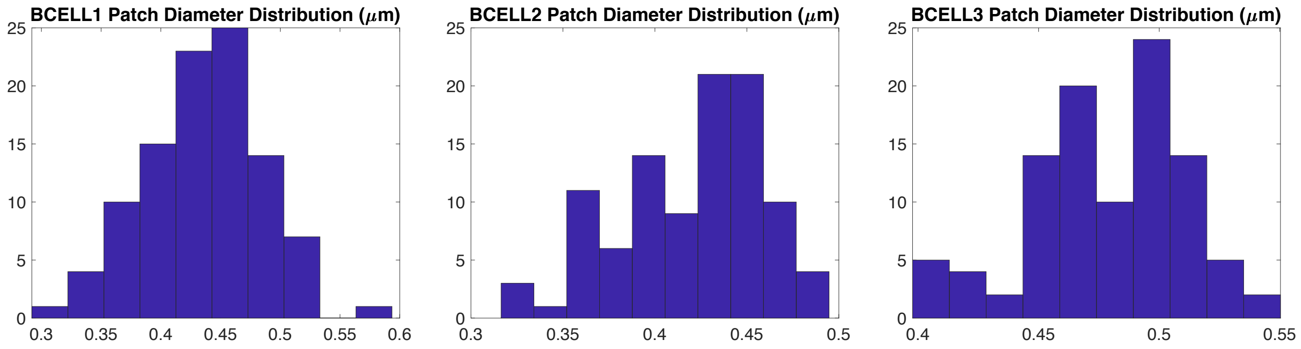

FIG. C: Distribution of patch diameters for the 100 patches in Bcell1, Bcell2 and Bcell3. Here diameter corresponds to the largest Euclidean distance between the center of two voxels within the patch.

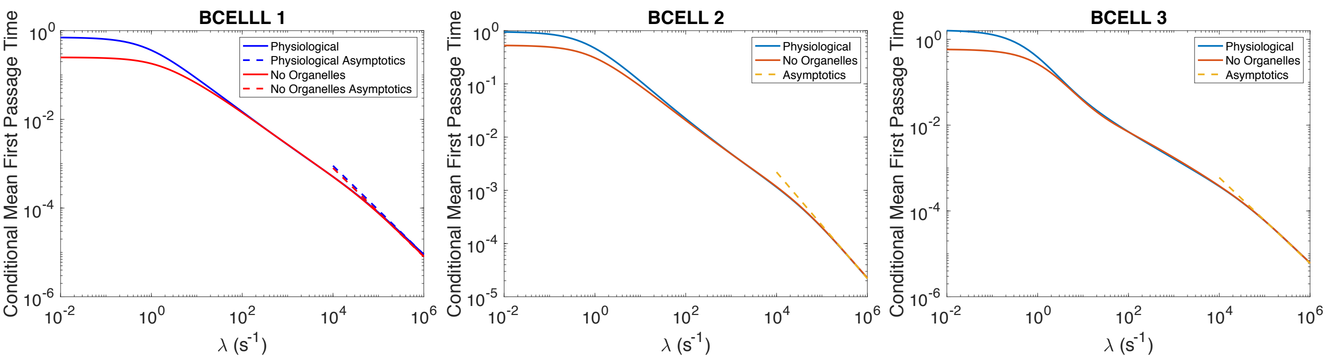

FIG. D: Convergence of $\langle T_{\lambda,h}\rangle$ to the asymptotic limit (17) as $\lambda \to \infty$ when the molecule is started uniformly on the surface of the cell. In Bcell1, the geodesic distance from the cell membrane to the nucleus is different in the "physiological" and "no organelles" cases, while in Bcell2 and Bcell3 the distance is the same (and so only one asymptotic line is shown).

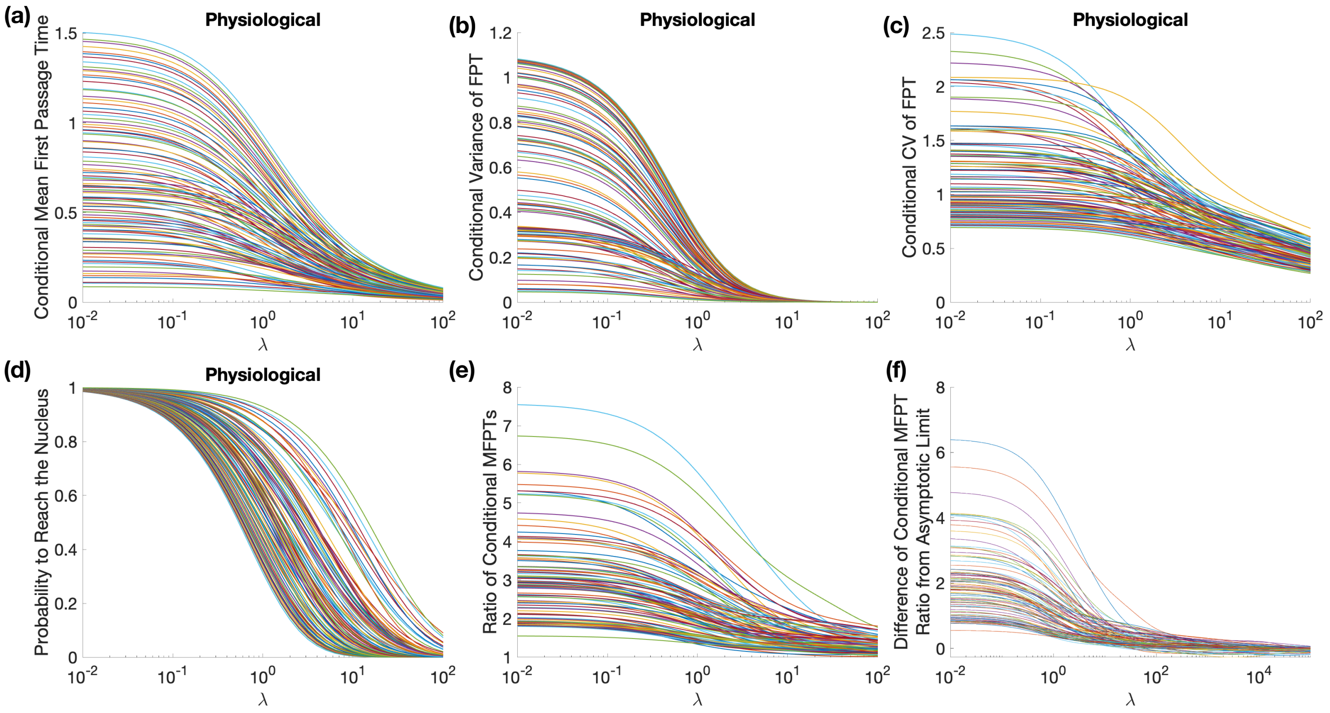

FIG. E: Statistics of the conditional MFPT, $T_{\lambda,h}$, for Bcell1 for 100 different patch initial conditions (see Section SI2). (a) through (d) show statistics for the physiological case. (e) shows the ratio of the physiological to no organelle conditional MFPTs, while (f) shows the difference between this ratio and the asymptotic limit.

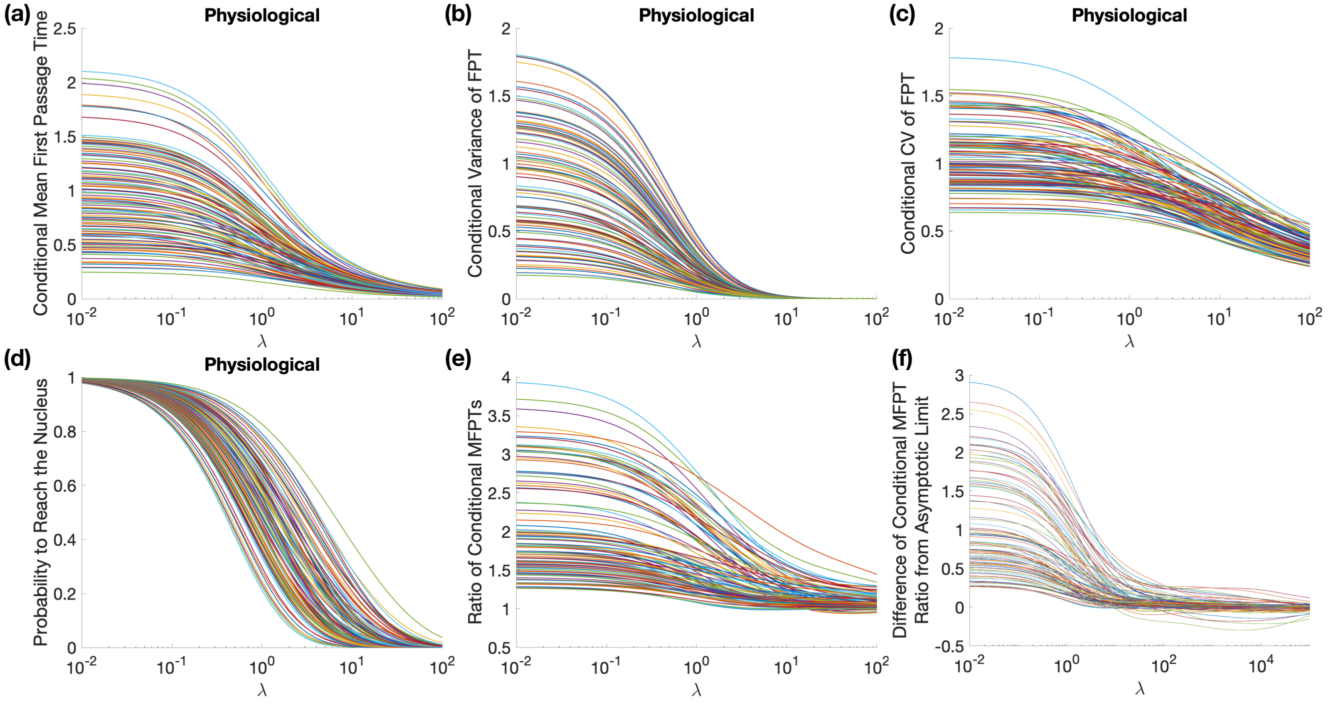

FIG. F: Statistics of the conditional MFPT, $T_{\lambda,h}$, for Bcell2 for 100 different patch initial conditions (see Section SI2). (a) through (d) show statistics for the physiological case. (e) shows the ratio of the physiological to no organelle conditional MFPTs, while (f) shows the difference between this ratio and the asymptotic limit.

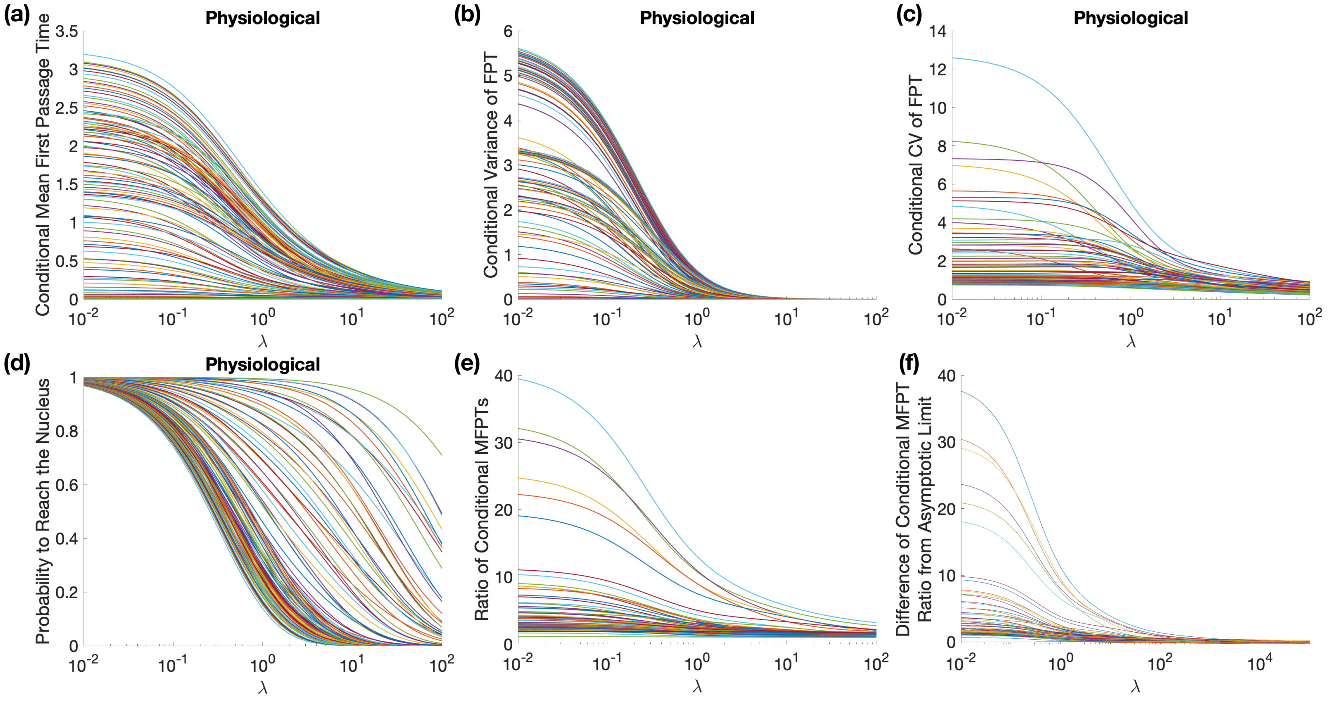

FIG. G: Statistics of the conditional MFPT, $T_{\lambda,h}$, for Bcell3 for 100 different patch initial conditions (see Section SI2). (a) through (d) show statistics for the physiological case. (e) shows the ratio of the physiological to no organelle conditional MFPTs, while (f) shows the difference between this ratio and the asymptotic limit.

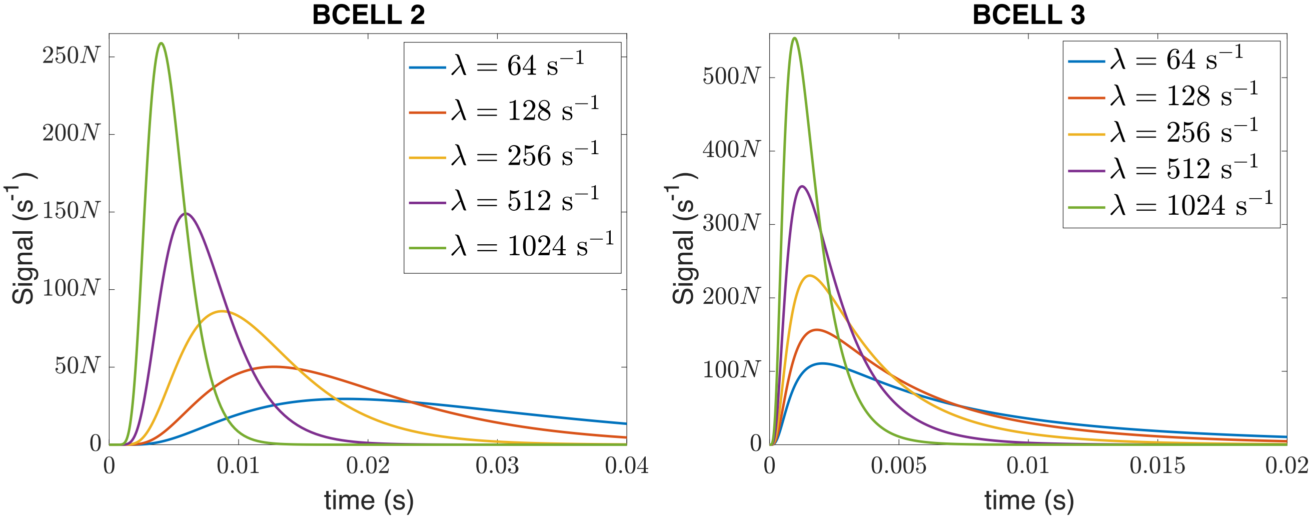

FIG. H: Signal successfully reaching the nucleus in Bcell2 and Bcell3. See Fig 4 in the main text for details.

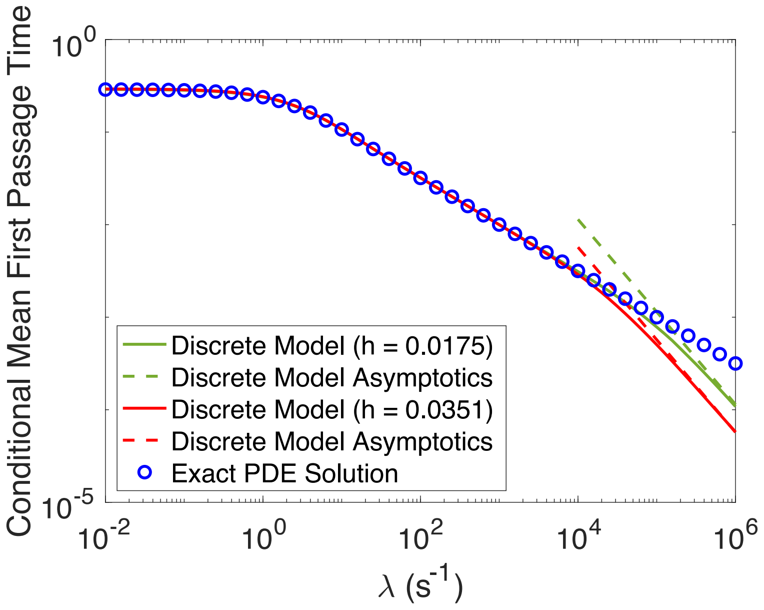

FIG. I: Conditional mean first passage time to reach the nucleus, $\langle T_\lambda \rangle = \mathbb{E}[T_\lambda | T_\lambda < \infty]$, when the nucleus is a sphere of radius 3μm, the cell membrane is a sphere of radius 5μm, and the cytosolic space between them is open (no organelle barriers). The figure shows the exact solution $\langle T_\lambda \rangle$ from the corresponding diffusion equation PDE (blue spheres), obtained by taking the logarithmic derivative of $F_{\lambda,0}(R)$, see Section SI3. The solid red line gives the numerical solution $\langle T_{\lambda,h} \rangle$ to the corresponding semi-discrete model using a Cartesian grid approximation to the cytosol with mesh spacing $h = 0.0351$ (comparable to the resolution of our B cell reconstructions). The green line gives the corresponding curve when the mesh spacing is reduced to $h = .0175$. Dashed lines give the asymptotic formula for the large λ behavior of $\langle T_{\lambda,h} \rangle$ (17). We see the continuous and discrete models agree very well until the asymptotic behavior takes over, demonstrating the different short time behavior of the underlying diffusion equation and semi-discrete diffusion equation solutions. As the mesh is refined, we also see that the semi-discrete model approximates the analytical value well to a larger value in λ, reflecting that the short-time breakdown of the approximation of the PDE by the semi-discrete model occurs on a shorter time-scale as the mesh is refined. See Section SI3 for details on the analytical solution and numerical simulations.

REFERENCE

1. Szabo A, Schulten K, Schulten Z. First passage time approach to diffusion controlled reactions. The Journal of Chemical Physics. 2008;72(8):4350–4357.
